# Microfluidic Spinning Boosting Thermoelectric Performance of PEDOT:PSS Nonwoven Fabrics

**DOI:** 10.1007/s40820-026-02227-3

**Published:** 2026-05-20

**Authors:** Yuhui Zhang, Hui Qiu, Jian Yang, Pengle Cao, Yu Wang, An-Quan Xie, Ke-Qin Zhang, Xiao-Qiao Wang

**Affiliations:** National Engineering Laboratory for Modern Silk, College of Textile and Clothing Engineering, Soochow University, Suzhou, 215123 People’s Republic of China

**Keywords:** PEDOT:PSS, Microfluidic spinning, Thermoelectric fabrics, Radiative cooling, Wearable electronics

## Abstract

Organic thermoelectric generators hold great promise for powering wearable microelectronics, yet their performance is fundamentally constrained by the trade-off between electrical conductivity (*σ*) and the Seebeck coefficient (*S*). Herein, we develop a microfluidic spinning platform to fabricate PEDOT:PSS-based nonwoven fabrics with precisely engineered micro-/nanoscale physical and electronic structures, substantially enhancing thermoelectric performance. The intense shear field and in situ coagulation within microfluidic microchannels, synergized with H_2_SO_4_ treatment, promotes axial orientation and coil-to-linear conformational transition of PEDOT chains, achieving multiscale structural ordering for highly efficient charge transport in the resulting fibers. A subsequent controlled NaOH‑mediated dedoping process finely tunes the Fermi level and modulates energy‑dependent scattering, yielding a final *σ* of 2038 S cm^−1^ and an *S* of 29.7 μV K^−1^. Such integrated modulation enables effective optimization of the classic *σ*-*S* trade-off, ultimately yielding a power factor of 179.8 μW m^−1^ K^−2^. Furthermore, by integrating the fabric with an electrospun PVDF-HFP radiative-cooling layer, we demonstrate a radiation-modulated fabric device capable of maintaining an in-plane temperature gradient (Δ*T* ≈ 20 K) under natural sunlight and efficiently harvesting ambient solar-thermal energy. This study provides a versatile route for the fabrication of all-organic, flexible fabrics with high-performance thermoelectric functionality for wearable energy applications.

**Supplementary Information:**

The online version contains supplementary material available at 10.1007/s40820-026-02227-3.

## Highlights

- A microfluidic spinning strategy is developed to fabricate PEDOT:PSS fibers with precisely engineered micro /nanoscale structures, effectively optimizing the trade-off between electrical conductivity and Seebeck coefficient.
- The PEDOT:PSS fiber achieves a high conductivity of 2038 S cm^−1^ and a Seebeck coefficient of 29.7 μV K^−1^, yielding a remarkable power factor of 179.8 μW m^−1^ K^−2^ among organic thermoelectric fibers.
- PEDOT fiber-based radiation-modulated nonwoven fabric device generates an in-plane temperature gradient of 20 K under sunlight (0.84 sun), enabling continuous solar thermoelectric energy harvesting for powering wearable microelectronics.

**Supplementary Information:**

The online version contains supplementary material available at 10.1007/s40820-026-02227-3.

## Graphical abstract

**Figure:**
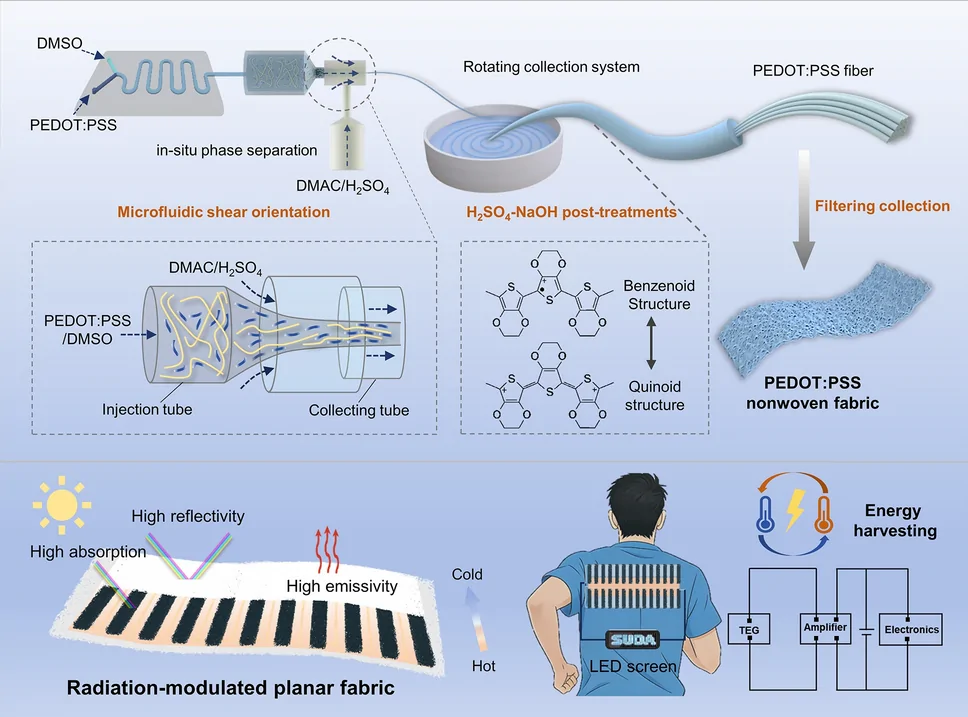

## Introduction

The rapid advancement of wearable electronics continues to drive the demand for sustainable and portable power solutions [1, 2]. Thermoelectric conversion technology that harnesses the Seebeck effect to harvest body heat or environmental waste heat offers a promising pathway toward self-powered wearable devices [3–5]. Fiber-based one-dimensional thermoelectric generators (f-TEGs) exhibit unique advantages over three-dimensional bulk and two-dimensional thin-film materials for wearable integration [6–8]. Their inherent flexibility ensures conformal contact with complex surfaces and seamless integration into textiles, while their high structural designability greatly expands the design possibilities for functional fabrics [9]. Unlike thin films that require additional supporting substrates, fibers can be directly integrated into textiles at the yarn level, preserving the fabric’s inherent breathability while effectively reducing overall weight and volume, and simultaneously exhibiting excellent mechanical durability. Based on the material composition, thermoelectric fibers can be classified into inorganic, organic, and inorganic–organic hybrid types [10]. Among these, organic thermoelectric fibers have attracted extensive research interest due to their lightweight nature, high flexibility, good biocompatibility, and relatively simple processing. The overall performance of thermoelectric materials is generally evaluated by the dimensionless figure of merit (*ZT* = *S*^2^*σT*/*κ*), where* S*, *σ*, *κ*, and *T* denote the Seebeck coefficient, electrical conductivity, thermal conductivity, and absolute temperature, respectively [11, 12]. The term *S*^2^*σ* is defined as the power factor (*PF*), which serves as a crucial parameter for assessing thermoelectric performance [13]. Given that organic thermoelectric materials generally have low thermal conductivity, improving their power factor has become the primary research focus for enhancing overall performance [14].

Among flexible organic thermoelectric materials, poly(3,4-ethylenedioxythiophene):poly(styrene sulfonate) (PEDOT:PSS) has gained considerable attention owing to its solution processability, biocompatibility, and chemical stability [15–17]. However, efforts to enhance the thermoelectric properties of PEDOT:PSS are constrained by two major issues: its disordered molecular arrangement and weak interchain coupling hinder charge transport, limiting electrical conductivity [18, 19], while optimizing performance is complicated by the inherent trade-off between electrical conductivity and the Seebeck coefficient, along with the complex interplay between molecular structure and transport mechanisms [20]. Recent studies on 2D PEDOT:PSS thin-film materials demonstrate that the control of carrier transport and doping level via delicate microstructure engineering is essential for coordinating electrical conductivity and the Seebeck coefficient. For example, Xu et al. sequentially treated PEDOT:PSS films with H_2_SO_4_, water, and a Tetrakis(dimethylamino)ethylene (TDAE) ethanol solution, combining secondary doping and dedoping processes to synergistically enhance the power factor to 526 μW m^−1^ K^−2^ [21]. In terms of fiber fabrication, solution-based wet spinning represents the most widely adopted route for producing PEDOT:PSS fibers. For example, Wu et al. reported a rapid and cost-effective wet-spinning method combined with DMSO doping and concentrated H_2_SO_4_ post-treatment to fabricate PEDOT:PSS fiber bundles, achieving a maximum power factor of 80.8 μW m^−1^ K^−2^ [22]. Similarly, Chen et al. fabricated PEDOT:PSS/1-ethyl-3-methylimidazolium dicyanamide (EMIM:DCA) composite fibers via wet spinning followed by a sulfuric acid immersion-stretching process, attaining a power factor of 85.5 μW m^−1^ K^−2^ [23]. However, wet spinning, relying on rapid phase separation and solidification, primarily focuses on polymer processing and scalable fiber shaping, lacking the precision required to control the physical and electronic microstructure of functional polymers at the micro- and nanoscale. This limitation results in the thermoelectric performance of existing PEDOT:PSS fibers remaining significantly inferior to their 2D counterparts.

Microfluidic spinning has recently emerged as a powerful platform for nanoprecision fiber production and structural engineering [24–26]. Unlike conventional wet spinning, microfluidic spinning enables continuous, one-step fabrication with precise flow control, allowing straightforward scale-up via parallelization of multiple microchannels. The well-defined shear field inside fluidic microchannels can induce targeted assembly and alignment of micro-/nanomaterials, thereby enabling the bottom-up programming of highly ordered, anisotropic structures from the molecular level upwards [27]. For example, He et al. employed a dual-scale spatially confined spinning technique to realize highly oriented co-assembly of 2D MXene nanosheets and 1D carbon nanofibers, establishing a tailored microstructure that supports outstanding electromechanical performance [28]. The microfluidic precise control over nanoscale assembly and in situ interactions holds significant potential for designing the physical and electronic microstructure of thermoelectric materials.

In parallel, passive thermal management strategies leveraging photothermal conversion and radiative cooling have emerged as effective approaches to enhance thermoelectric generation by establishing temperature gradients without external energy input [29–31]. For example, Hou et al. developed a microencapsulated phase change material (MPCM) with an n-docosane core and a TiO_2_/Ti_2_O_3_ composite shell [32]. By integrating a polydimethylsiloxane/MPCM composite film with a thermoelectric module, they harnessed photothermal conversion, energy storage, and radiative cooling to achieve continuous 24-h power generation. Notably, vertical device architectures face inherent limitations for textile-based thermoelectric applications. Spatial overlap between the radiative cooler and solar absorber in such configurations can hinder stable temperature gradient formation [33]. Moreover, due to the finite thickness of fabrics, establishing a significant temperature gradient is challenging. While increasing the thickness of the device could theoretically enhance Δ*T*, this approach conflicts with the essential requirements of wearable comfort. In contrast, planar configurations avoid mutual shading by laterally arranging the hot and cold ends, enabling simultaneous access to solar heating and radiative cooling. Fiber-based TEGs offer an ideal platform for realizing planar-architecture thermoelectric devices.

Herein, we report a microfluidic spinning strategy for fabricating high-performance PEDOT:PSS nonwoven fabrics. The shear-induced axial orientation and extension of PEDOT chains during microfluidic spinning, coupled with H_2_SO_4_ treatment, construct highly ordered conductive pathways and optimizes the doping state, maximizing the fiber electrical conductivity to be 2857 S cm^−1^. Independently, a controlled NaOH-mediated dedoping process precisely tunes the Fermi level and energy-dependent scattering, maximizing *S* with low loss in conductivity. The resulting wearable TEG achieves a high power factor of 179.8 μW m^−1^ K^−2^. Furthermore, by integrating a PEDOT:PSS fiber-based nonwoven fabric with an electrospun PVDF-HFP radiative-cooling layer, we fabricate a novel radiation-modulated planar fabric. The spatially designed photothermal conversion and passive radiative cooling of this fabric establish a stable in-plane temperature gradient (Δ*T* ≈ 20 K) under natural sunlight (0.84 kW m^−2^), enabling direct solar-to-thermoelectric power generation.

## Experimental Section

### Materials

The poly(3,4-ethylenedioxythiophene):poly(styrene sulfonate) (PEDOT:PSS) aqueous dispersion (Clevios PH1000) was purchased from Heraeus. Dimethyl sulfoxide (DMSO, AR) was obtained from Shanghai Macklin Biochemical Co., Ltd. Dimethylacetamide (DMAc, AR) and N,N-dimethylformamide (DMF, AR) were supplied by Shanghai Aladdin Biochemical Technology Co., Ltd. Acetone (AR) was also acquired from the same supplier (Shanghai Aladdin). Sulfuric acid (H_2_SO_4_, 98%, AR) was purchased from Shanghai Lingfeng Chemical Reagent Co., Ltd. Sodium hydroxide (NaOH, AR) was acquired from Sinopharm Chemical Reagent Co., Ltd. (Shanghai, China). Poly(vinylidene fluoride-co-hexafluoropropylene) (PVDF-HFP, AR) was purchased from Sigma-Aldrich.

### Fabrication of PEDOT:PSS Nonwoven Fabrics

The PEDOT:PSS nonwoven fabric was fabricated via a microfluidic spinning strategy. Specifically, 10 mL of PEDOT:PSS aqueous dispersion (initial pH = 2.6) and 0.5 mL of DMSO additive were separately injected into the two inlet channels of a Y‑shaped microfluidic device for rapid in‑channel mixing. The blended stream was subsequently introduced into the shear field of a DMAc/H_2_SO_4_ mixture, with a volume ratio of 1:1 and H_2_SO_4_ concentration of 1 M, within a confinement tube. The microfluidic spinning setup consists of a coaxial microchannel with an inner diameter of 0.6 mm. The shear rate at the core–sheath interface is governed by the velocity gradient, which is determined by the flow rates and the channel dimensions. Detailed microfluidic parameters are provided in Supporting Information Table S1. The resulting gel fibers were collected on a sieve. The collected fibers then underwent sequential chemical post‑treatments, beginning with H_2_SO_4_ immersion. After thorough rinsing with deionized water to remove residual acid, the fibers were subjected to NaOH treatment (25 °C, 0.5 h) under static immersion conditions. Following NaOH treatment, the fibers were initially rinsed with deionized water, yielding a rinse water pH of approximately 10–11 due to residual alkali. Rinsing was continued until the rinse water reached a stable neutral pH of 7. Finally, the fibers were dried at 60 °C under pressure to obtain a nonwoven fabric characterized by numerous fused junctions.

### Fabrication of a Radiation-Modulated Planar Fabric

A white polyester fabric was used as the flexible substrate. The PEDOT:PSS fabric was fixed onto the polyester substrate using a PDMS binder. Subsequently, the radiation-modulated planar fabric was fabricated by directly electrospinning the PVDF-HFP membranes onto the PEDOT:PSS fabric. PVDF‑HFP membranes were fabricated as radiative-cooling layers through an electrospinning process. First, a homogeneous spinning dope was prepared by dissolving 4 g of PVDF‑HFP particles in a binary solvent system consisting of 14 g N,N-dimethylformamide (DMF) and 6 g acetone. The mixture was magnetically stirred at room temperature until complete dissolution. Electrospinning was conducted using an infusion pump to deliver the solution at a controlled feed rate of 0.5 mL h^−1^ and DC voltage of 20 kV.

### Characterization

Surface morphology and electrical mapping were characterized by atomic force microscopy and conductive-atomic force microscopy (AFM/C-AFM, Dimension Icon, Bruker, USA). For fiber samples, individual microfibers were carefully deposited onto freshly cleaved mica substrates and fixed at both ends with silver paste to ensure stable contact and minimal movement during scanning. All measurements were conducted in tapping mode under ambient conditions. Silicon cantilevers with a nominal resonance frequency of 75 kHz and a spring constant of 2.8 N m^−1^ were used for topography imaging. For C-AFM measurements, conductive Pt/Ir-coated tips were employed, and a DC bias of 50 mV was applied between the tip and the sample during current mapping. Small-angle X-ray scattering (SAXS) and wide-angle X-ray scattering (WAXS) measurements were performed using Cu Kα radiation (λ = 1.5418 Å, Xeuss 3.0, Xenocs, France). Scanning electron microscopy (SEM) characterization was performed using a field-emission instrument (Regulus 8230, Hitachi, Japan). X-ray photoelectron spectroscopy (XPS) analysis was conducted on an Axis Ultra HAS spectrometer (Shimadzu, Japan). X-ray diffraction (XRD) patterns were collected on an XRD-6100 diffractometer (Shimadzu, Japan). UV–Vis absorption spectra were recorded using a UV-3600 spectrophotometer (Shimadzu, Japan). Raman spectroscopy measurements were performed on a HORIBA XploRA PLUS system (France). Ultraviolet photoelectron spectroscopy (UPS) analysis was conducted on a Thermo ESCALAB 250XI system. Mechanical properties were tested using a universal testing system (HY-0580, Hengyi, China) at a constant loading rate of 30 mm min^−1^. Interfacial adhesion between the PVDF-HFP layer and the PEDOT:PSS fabric was evaluated by 180-degree peel tests using the same system at a peeling rate of 300 mm min^−1^. The electrical properties were measured on a four-probe resistivity meter (RTS-11, China). The Seebeck coefficient was measured by applying a temperature gradient (Δ*T*) across the sample using a thin-film heater, while simultaneously recording the induced thermoelectric voltage (Δ*V*). For the thermoelectric measurements, at least five samples from the same batch of fibers were tested, and the error bars represent the standard deviation between these samples.

## Results and Discussion

### Schematic Illustration of the Microfluidic Spinning Strategy for Fabricating Thermoelectric PEDOT:PSS Nonwoven Fabrics

**Fig. 1:**
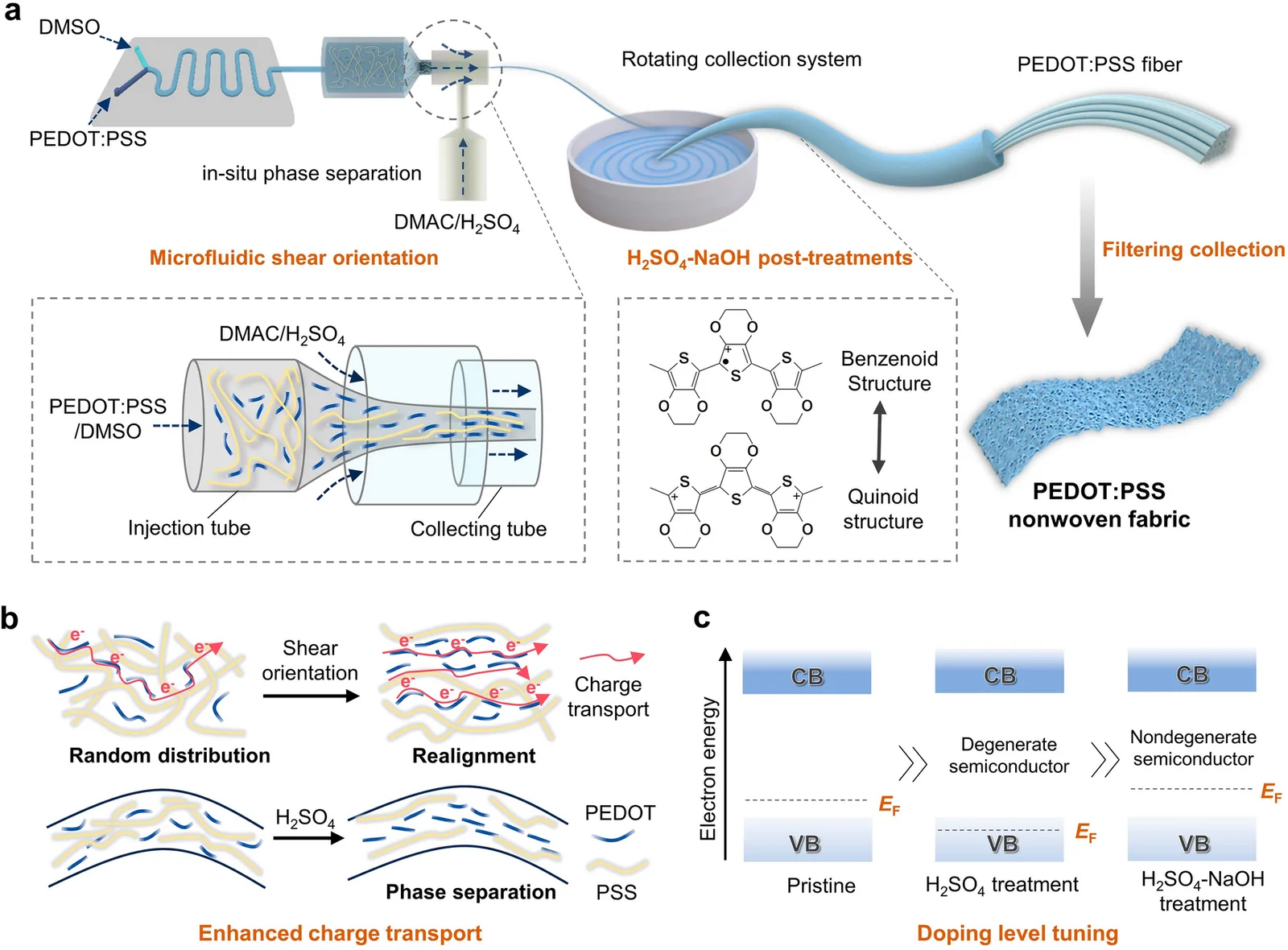
Schematic illustration of the microfluidic spinning strategy for fabricating thermoelectric PEDOT:PSS nonwoven fabrics.** a** Fabrication process of PEDOT:PSS nonwoven fabrics. **b** Enhancement of charge transport in PEDOT:PSS fibers through shear-induced alignment and acid treatment for extension of PEDOT chains. **c** Doping-level tuning via base treatment for the Seebeck coefficient enhancement

The microstructure of PEDOT:PSS fibers was precisely engineered through shear flow control within a microfluidic device (Fig. 1a). Specifically, a Y-shaped microchannel was employed to homogeneously blend a DMSO additive (5% v/v) into the PEDOT:PSS aqueous dispersion. Within this confined geometry, intense chaotic advection facilitates rapid mixing and intimate interaction between DMSO molecules and the PEDOT:PSS chains. The mixed dope was then injected into a coaxial‑flowing sheath stream consisting of a DMAc/H_2_SO_4_ mixture. The high viscosity ratio between the core and sheath flows generated strong laminar shear at the interface, stretching and aligning the PEDOT:PSS chains along the flow direction. Unlike rapid coagulation, the DMAc/H_2_SO_4_ system promoted a transient gel state, providing a suitable time window for full chain alignment [34]. This approach enabled the synchronous coupling of shear-induced alignment and controlled phase separation in PEDOT:PSS during solidification, yielding fibers with anisotropic microstructures and minimal defects. The resulting highly oriented structure shortened the pathways for charge-carrier transport along the fiber axis, leading to a large enhancement in electrical conductivity. Further conductivity improvement was achieved through H_2_SO_4_ treatment, which modifies the structure via a conformational transition of PEDOT chains from coiled to linear and the partial removal of insulating PSS (Fig. 1b). These synergistic changes optimized the charge-transport network, yielding a significant boost in conductivity. The Seebeck coefficient was specifically enhanced by a subsequent base treatment. According to the relation *S* ∝|*E*_T_* − E*_F_|/*kT* (where *E*_T_ is the mean carrier energy, *E*_F_ is the Fermi level, *k* is the Boltzmann constant, and *T* is the absolute temperature), the Seebeck coefficient can be enhanced by tuning the Fermi level (*E*_F_) [35, 36]. The preceding acid treatment places the material in a heavily doped state, with *E*_F_ deep inside the valence band, resulting in a low Seebeck coefficient. The subsequent NaOH treatment then induces a chemical dedoping process, which reduces the carrier concentration and thereby shifts *E*_F_ out of the valence band. This transition converts the material into a nondegenerate semiconductor state, effectively enhancing the Seebeck coefficient (Fig. 1c). The prepared PEDOT:PSS fibers were collected and processed via filtration into nonwoven fabrics, forming a continuous and densely-packed fibrous network.

### Characterization of Micro-orientation in PEDOT:PSS Fibers Fabricated at Different Shear Rates

**Fig. 2:**
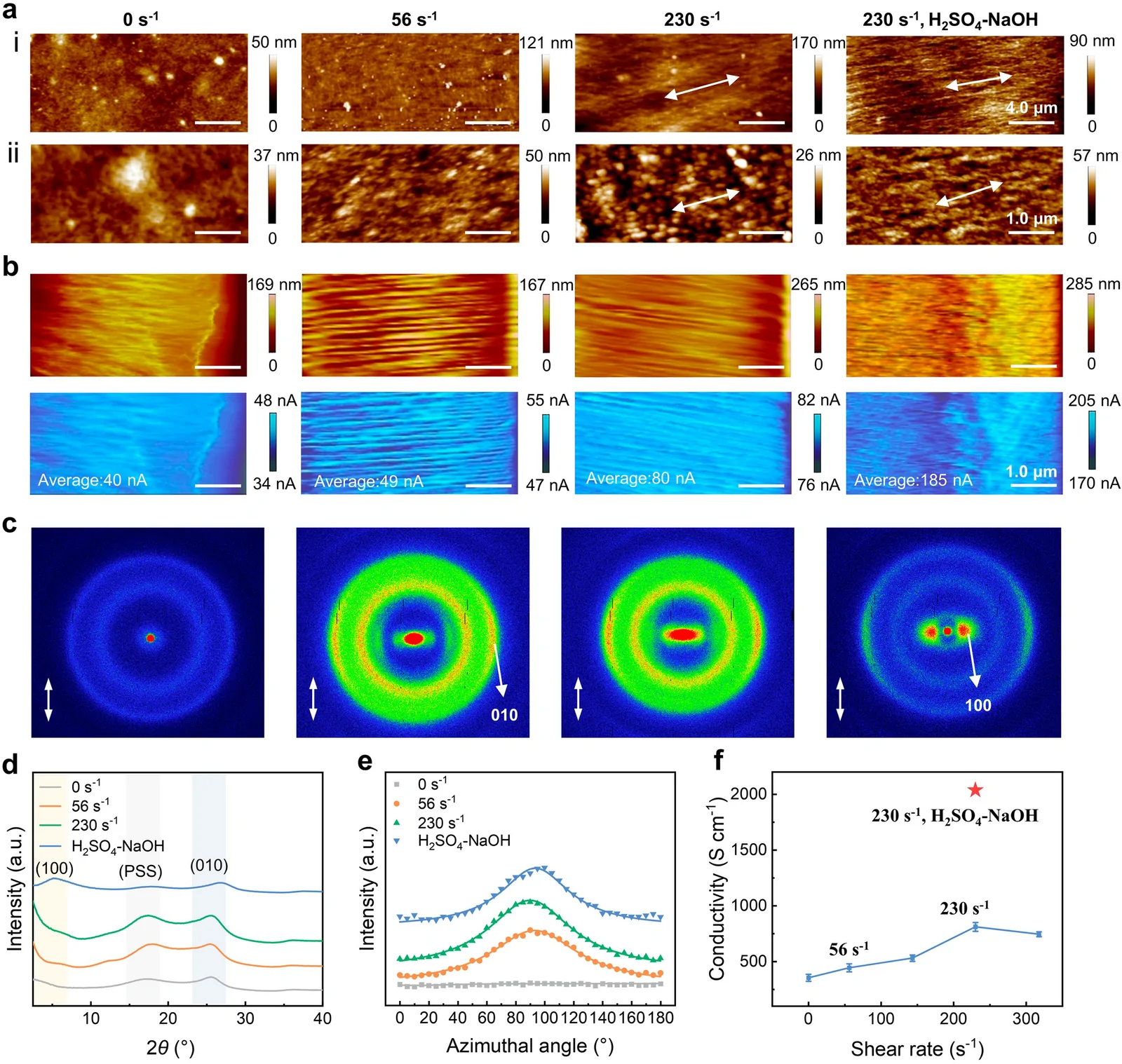
Characterization of micro-orientation in PEDOT:PSS fibers fabricated at different shear rates. **a** AFM images, **b** C-AFM images and current mapping images, **c** 2D WAXS patterns, **d** 1D WAXS profiles, and **e** azimuthally dependent scattering spectra (*q* = 1.79 Å^−1^) for a drop-cast film (0 s^−1^), fibers sheared at 56 and 230 s^−1^, and the 230 s^−1^ fiber after H_2_SO_4_-NaOH treatment. The double-headed arrows in the images denote the fiber axial direction. **f** Conductivity of microfluidic-spun PEDOT:PSS fibers at different shear rates

The micromorphology of PEDOT:PSS fibers is predominantly governed by the shear flow field during the microfluidic spinning process. The shear rate (*D*), defined as *D* = d*v/*d*x* (where d*v* is the velocity differential between adjacent fluid layers, and d*x* is the radial distance increment from the channel wall), serves as the key parameter quantifying the shear effect and directly regulates the formation of micro‑/nanoscale ordered structures within the fiber. Higher shear rates promote pronounced alignment of the polymer chains along the flow direction, driving a structural transition from a disordered to an ordered state. Morphological evolution across varying shear regimes was systematically characterized via atomic force microscopy (AFM) (Figs. 2a and S1). The drop-cast film, prepared at a near‑zero shear rate (0 s^−1^), exhibited a nanoscopically disordered morphology. In contrast, microfluidic spinning induced a distinct transition toward uniaxial order. At a modest shear rate (56 s^−1^), PEDOT molecular chains began to align along the fiber axis, signaling the onset of structural anisotropy. When the shear rate increased to 230 s^−1^, this alignment was significantly enhanced, resulting in highly ordered nanofibrillar architecture. This shear-induced orientation process, accompanied by in situ phase separation, promoted the formation of continuous nanoconductive pathways, establishing a critical structural foundation for high electrical conductivity. Notably, in the untreated sample spun at 230 s^−1^, PEDOT chains retained a coiled conformation, with high-resolution AFM images displaying aligned spherical granules. After post-treatment, the granular structures within the sample were reduced, transforming into an extended, fibrous network [37–39]. To correlate these morphological refinements with electronic transport behavior, we employed conductive-atomic force microscopy (C-AFM) for spatially resolved current mapping (Fig. 2b). The PEDOT:PSS film exhibited substantial electrical inhomogeneity, indicating constrained charge-transport capability. In contrast, PEDOT:PSS fibers prepared via high-shear-rate spinning displayed denser and more uniform current distribution. These observations demonstrate that shear-induced alignment during processing leads to the formation of an interconnected conductive pathway, which enhances the overall electrical conductivity of the material. Molecular orientation and crystalline structure were further characterized using wide-angle X-ray and small-angle scattering (WAXS/SAXS). Two-dimensional WAXS patterns (Fig. 2c) illustrate molecular-scale orientation. The drop-cast film displayed nearly uniform diffraction rings, confirming isotropic structure with random PEDOT chain orientation, while the fiber samples showed characteristic diffraction arcs, demonstrating preferred crystal orientation along the fiber axis [40]. The PEDOT crystal structure is schematically illustrated in Fig. S2, where the (100) plane corresponds to lamellar stacking with alternating PEDOT and PSS chains, while the (010) plane represents π-π stacking [41]. Figure 2d shows the normalized X-ray intensity versus diffraction angle (2*θ*). The broad scattering peak at approximately 17.4° was significantly reduced after post-treatment. This peak primarily originated from amorphous and randomly oriented PSS, and its attenuation confirms effective removal of insulating PSS components via acid treatment. The (010) diffraction peak shifted toward higher *q*-values, indicating reduced π-π stacking distance [42]. This result demonstrates that shear-induced orientation combined with chemical treatment promotes tighter π-π stacking of PEDOT molecules, creating favorable conditions for efficient interchain charge transport. To quantitatively evaluate the orientation degree, azimuthal scanning was performed on the diffraction peak at *q* = 1.79 Å^−1^ (Fig. 2e). The full width at half maximum (FWHM) decreases progressively with increasing spinning shear rate, while the orientation factor rises correspondingly to 0.61 at 230 s^−1^, confirming improved molecular alignment along the fiber axis. Post-treatment further reduced the FWHM, yielding an orientation factor of 0.68, which demonstrates its positive effect on orientation refinement. Two-dimensional SAXS patterns revealed consistent results. As the shear rate increased, the orientational order of the nanostructures was significantly enhanced (Figs. S3 and S4). In the 230 s^−1^ fiber after H_2_SO_4_-NaOH treatment, a scattering maximum was observed at *q* ≈ 0.38 Å^−1^ (Fig. S5), corresponding to a domain spacing of approximately 16.5 Å resulting from the phase-separated structure of PEDOT:PSS. The improved orientation and extension of PEDOT:PSS chains directly enhance the electrical conductivity of the resulting fibers. The electrical conductivity increased from 355 to 812 S cm^−1^ as the shear rate increased from 0 to 230 s^−1^ (Fig. 2f). After H_2_SO_4_-NaOH post-treatment, the conductivity was further enhanced to 2038 S cm^−1^.

### Microstructure and Physicochemical Characterizations of the PEDOT: PSS Fabric

**Fig. 3:**
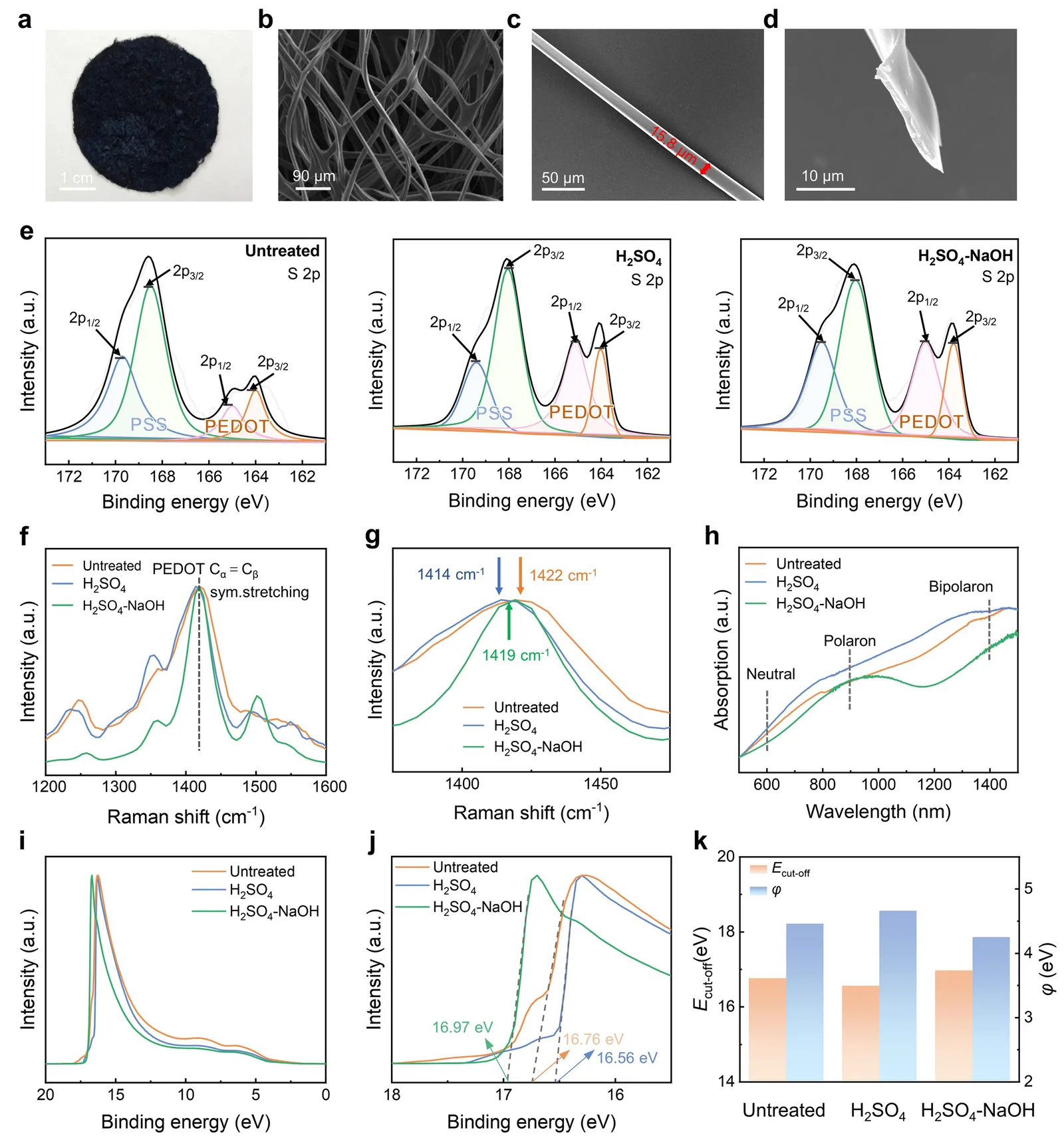
Microstructure and physicochemical characterizations of the PEDOT:PSS fabric. **a** Photograph and **b** surface SEM image of the H_2_SO_4_-NaOH-treated PEDOT:PSS fabric. **c** SEM image and **d** cross-sectional SEM image of a single fiber. **e** S 2*p* XPS spectra, **f** Raman spectra, **g** zoom-in spectra for the wavenumbers ranging from 1375 to 1475 cm^−1^. **h** UV–Vis–NIR absorption spectra, **i** UPS spectra, **j** the cut-off edge, and **k** the cut-off energy and the work function of the untreated, H_2_SO_4_-treated, and H_2_SO_4_-NaOH-treated PEDOT:PSS fabrics

The macroscopic morphology and microstructure of the fabricated PEDOT:PSS nonwoven fabric were analyzed by scanning electron microscope (SEM) (Fig. 3a-d). The material assembles into a three-dimensional, porous network of tightly interwoven fibers, which affords the fabric high porosity, breathability, and mechanical flexibility. The fabric can be readily cut, folded, and bent without structural compromise (Fig. S6). To elucidate the fiber formation mechanism, we systematically investigated its morphological evolution. As-spun fibers exhibit a distinctive flat, ribbon-like cross section, resulting from an asymmetric phase separation process. During this process, dilute sulfuric acid rapidly protonated the PSS chains, inducing fast solidification of the fiber surface and forming a rigid skin layer. Meanwhile, DMAc acted as a good solvent, delaying phase separation in the core and keeping it in a plastic state. Subsequent solvent exchange induced continuous contraction of the plastic core, which is constrained by the outer rigid skin, leading to structural instability and eventual collapse into a ribbon-like cross section. Furthermore, at a shear rate of 56 s^−1^, the fiber length is approximately 86 μm, while at 230 s^−1^, it reduces to approximately 67 μm (Fig. S7). This reduction is attributed to the enhanced stretching effect of the sheath flow on the core fluid at higher shear rates. The influence of H_2_SO_4_ treatment time on fiber morphology was investigated, revealing that prolonged treatment induces significant axial shrinkage accompanied by radial expansion of the fibers (Figs. S8 and S9). X-ray photoelectron spectroscopy (XPS) was used to quantify the insulating PSS content based on the S 2*p* spectral features (Fig. 3e). The peaks at 162–166 and 166–172 eV correspond to sulfur in PEDOT and PSS, respectively [34, 43]. After H_2_SO_4_ treatment, the PEDOT/PSS peak area ratio increased from 23.5% to 65.1%, confirming effective removal of insulating PSS. Subsequent NaOH treatment induced no further change in this ratio, indicating that the dedoping process primarily modulates the oxidation state rather than the phase composition. The structural ordering induced by PSS removal was further corroborated by X-ray diffraction (XRD) (Fig. S10). The diffraction peak at approximately 6.4°, corresponding to the lamellar (100) stacking of alternating PEDOT and PSS chains, became notably sharper after post-treatment. This sharpening indicates that PSS removal significantly reduced structural disorder and enhanced crystallinity [44]. Raman spectroscopy was used to probe the chemical structure and conformational evolution of PEDOT chains (Fig. 3f, g). The signal between 1400 and 1500 cm^−1^ is attributed to C_α_ = C_β_ stretching vibrations in PEDOT [45]. Following H_2_SO_4_ treatment, a redshift to 1414 cm^−1^ indicated a conformational transition from coiled benzoid to linear quinoid structures, consistent with enhanced chain ordering [46, 47]. Subsequent NaOH treatment reversed the peak position to 1419 cm^−1^, confirming the reversible nature of the conformational change and demonstrating precise control over the PEDOT chain conformation via chemical post-treatments. The concurrent sharpening of the Raman peak upon NaOH treatment is consistent with the reported behavior of dedoped PEDOT:PSS, where neutral PEDOT segments exhibit a stronger Raman response under 514.53 nm excitation, further supporting a reduction in the oxidation level of PEDOT after dedoping [48]. To elucidate the enhancement mechanism of the Seebeck coefficient, this study combined UV–Vis–NIR absorption spectroscopy with ultraviolet photoelectron spectroscopy (UPS). According to established literature, the neutral state of PEDOT chains shows absorption at approximately 600 nm, the polaron state at 900 nm, and the bipolaron state at 1400 nm [49]. Compared to the untreated sample, the H_2_SO_4_-treated sample shows significantly enhanced absorption at both 900 nm and 1400 nm (Fig. 3h). This indicates a higher oxidation degree and an increased doping level, corresponding to a rise in charge-carrier concentration. After H_2_SO_4_-NaOH treatment, the absorption at 900 nm increased while the 1400 nm band decreased, suggesting that NaOH promotes the reduction of bipolarons to polarons. This leads to partial dedoping and a lower overall oxidation state, a trend confirmed by the reduced carrier concentration (Fig. S11). As the Seebeck coefficient typically increases while conductivity decreases with reduced carrier concentration, reducing carrier concentration provides a viable route to enhance the Seebeck coefficient, particularly if the concomitant loss in conductivity can be mitigated. UPS analysis further reveals the underlying electronic structure evolution (Fig. 3i-k). The work function (*φ*) was calculated using the formula *φ* = 21.22−*E*_cut-off_, where *E*_cut-off_ is the cut-off energy and 21.22 eV is the incident photon energy [35]. The *E*_cut-off_ values were determined by tangent fitting of the secondary electron cutoff edge. The measured *E*_cut-off_ values for the untreated, H_2_SO_4_-treated, and H_2_SO_4_-NaOH-treated sample are 16.76, 16.56, and 16.97 eV, respectively. The corresponding work functions are 4.46, 4.66, and 4.25 eV. This progression clearly reflects the evolution of the Fermi level (*E*_F_). The increased work function after H_2_SO_4_ treatment indicates a downward shift of *E*_F_ deeper into the valence band, corresponding to a lower Seebeck coefficient. In contrast, subsequent NaOH treatment significantly reduces the work function, indicating a pronounced upward shift of *E*_F_, which in turn increases the value of |*E*_T_−*E*_F_|. According to the Mott relation, where the Seebeck coefficient is linearly proportional to |*E*_T_−*E*_F_|, the increase in this energy difference therefore directly enhances the Seebeck coefficient.

### Thermoelectric and Mechanical Properties of PEDOT:PSS Fabrics

**Fig. 4:**
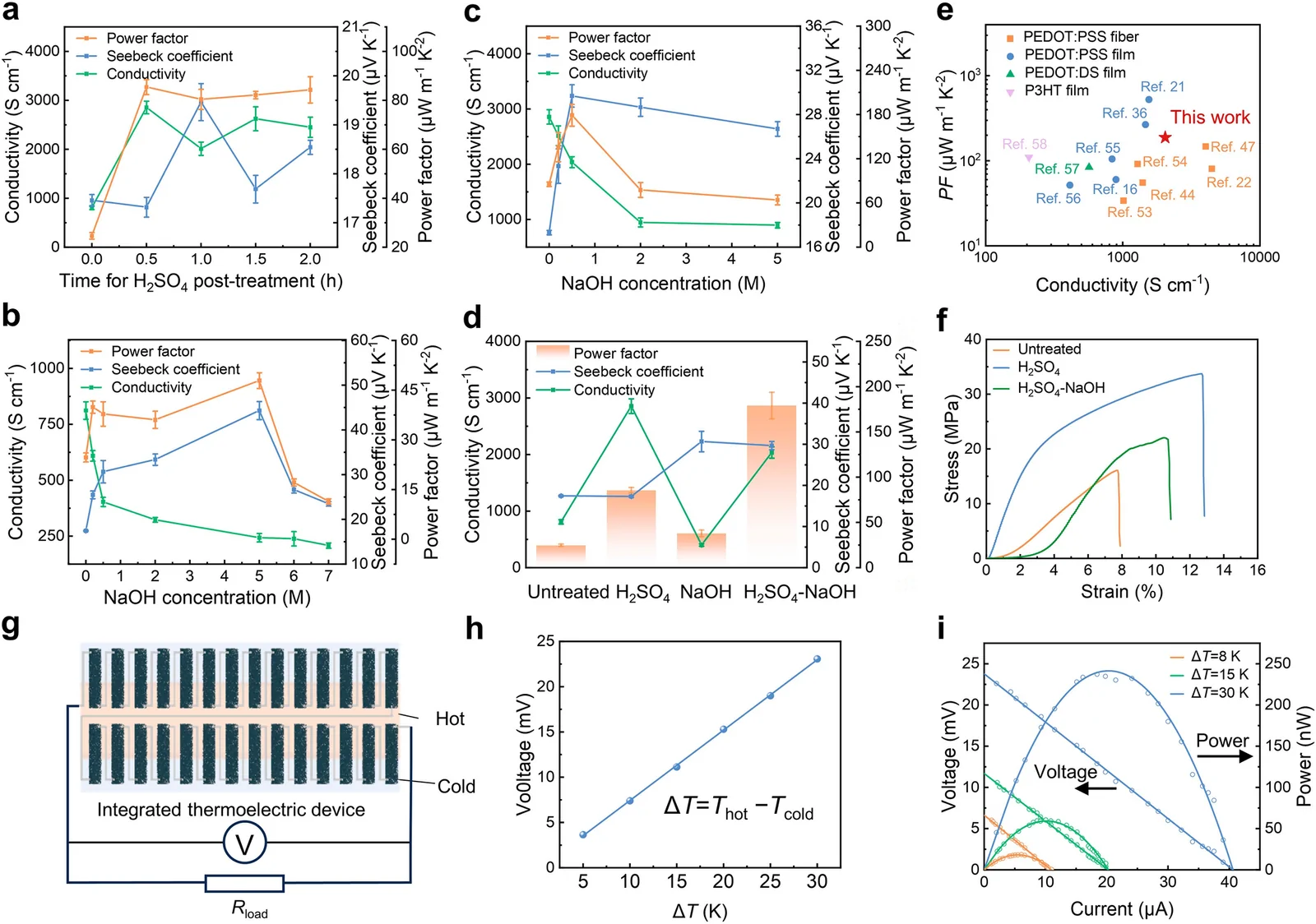
Thermoelectric and mechanical properties of PEDOT:PSS fabrics. **a** Thermoelectric property of H_2_SO_4_-treated fibers as a function of time for H_2_SO_4_ treatment. **b** Thermoelectric properties of NaOH-treated fibers as a function of NaOH concentration. **c** Thermoelectric properties of H_2_SO_4_-NaOH-treated fibers as a function of NaOH concentration. **d** Comparison of the optimal properties achieved by different post-treatment routes. **e** Comparison of the power factor achieved in this work with reported values for organic fibers and films [16, 21, 22, 36, 44, 47, 53–58]. **f** Mechanical properties of untreated, H_2_SO_4_-treated and H_2_SO_4_-NaOH-treated PEDOT:PSS fabrics. **g** Schematic of the flexible thermoelectric device comprising 24 treated fabric legs connected in series. **h** Open-circuit voltage as a function of the applied temperature difference (Δ*T*). **i** Output voltage and power as functions of current at Δ*T* = 8, 15, and 30 K

Building upon the structurally aligned foundation established through microfluidic spinning, we systematically investigated the impact of acid–base post-treatment on thermoelectric performance. The pristine, untreated PEDOT:PSS fiber exhibited a conductivity of 812 S cm^−1^ and a Seebeck coefficient of 17.5 μV K^−1^. The enhancement of charge transport was achieved through secondary doping with concentrated H_2_SO_4_. The electrical properties were critically influenced by the duration of H_2_SO_4_ immersion (Fig. 4a). Insufficient treatment led to incomplete reaction between H_2_SO_4_ and PEDOT:PSS, while excessive treatment may cause material over-oxidation [47]. The optimal H_2_SO_4_ post-treatment time was determined to be 0.5 h, yielding a conductivity of 2857 S cm^−1^ and a Seebeck coefficient of 17.3 μV K^−1^, with a corresponding power factor of 85.5 μW m^−1^ K^−2^. Independent tuning of the Seebeck coefficient was initially investigated through direct NaOH-mediated dedoping of the pristine, untreated sample. The voltage output of the NaOH-treated fiber showed a linear response to temperature gradients (Fig. S12). The electrical conductivity exhibited a monotonic decrease with increasing NaOH concentration, whereas the Seebeck coefficient displayed a nonmonotonic trend, characterized by initial increase to the peak followed by continuous decline (Fig. 4b). A maximum power factor of 47.9 μW m^−1^ K^−2^ was achieved at an optimal concentration of 5 M, with corresponding conductivity and Seebeck coefficient values of 243 S cm^−1^ and 44.4 μV K^−1^, respectively. Notably, the decrease in the Seebeck coefficient at excessively high NaOH concentrations is attributed to the disruption of the ordered PEDOT chain structure and reduced crystallinity, which hinders directional carrier transport [50]. Crucially, applying the NaOH treatment after the H_2_SO_4_ step enabled the optimization of *S* without sacrificing the high *σ* achieved initially. The fiber was first treated with H_2_SO_4_ (0.5 h) to establish a high-conductivity network, followed by immersion in NaOH solutions to finely tune the doping level. The thermoelectric properties exhibited a clear dependence on NaOH concentration (Fig. 4c), and the output voltage showed a linear response to the applied temperature gradient (Fig. S13). At the optimal NaOH concentration of 0.5 M, the fiber achieved a conductivity of 2038 S cm^−1^ and a Seebeck coefficient of 29.7 μV K^−1^, corresponding to a power factor of 179.8 μW m^−1^ K^−2^ (Fig. 4d). For comparison, drop-cast PEDOT:PSS films subjected to the same H_2_SO_4_-NaOH post-treatments achieved a power factor of only 23.1 μW m^−1^ K^−2^ (Fig. S14). Furthermore, PEDOT:PSS intrinsically exhibits low thermal conductivity, with values ranging from 0.17 to 0.37 W m^−1^ K^−1^ in the literature [51, 52]. For instance, PEDOT:PSS films treated sequentially with H_2_SO_4_ and NaOH show an in-plane thermal conductivity of approximately 0.35 W m^−1^ K^−1^ [35]. Based on this reported range of 0.17–0.37 W m^−1^ K^−1^, the *ZT* for the fiber at room temperature is estimated to be 0.15–0.32, further validating the effectiveness of our microfluidic spinning and post-treatment strategy. To ensure the reliability of the measured thermoelectric properties, all samples were thoroughly rinsed and dried to eliminate any potential interference from residual ions or moisture. Additionally, we carefully considered the potential effects of these residual species—which may introduce ionic thermodiffusion artifacts in the Seebeck coefficient measurements and compromise long-term stability through structural degradation. The success of this strategy validates the proposed concept of concerted modulation, wherein microfluidic shear combined with acid treatment for orientation and extension of PEDOT chains engineers an ordered and low-defect pathway for high electrical conductivity, while the subsequent mild base treatment fine-tunes the doping level to maximize the Seebeck coefficient. The resulting power factor surpasses that of most reported organic thermoelectric fibers (Fig. 4e and Table S2). Although the power factor remains lower than that of some 2D films fabricated by drop casting or spinning coating, our microfluidic spinning strategy is promising for scalable fiber fabrication, which is essential for wearable integration. Additionally, the post-treatments significantly increase the tensile strength of the PEDOT:PSS fabric to 22 MPa (Fig. 4f). The increased tensile strength results from the removal of insulating PSS, the conformational transition of PEDOT chains, and enhanced molecular orientation, which collectively facilitate efficient stress transfer. Coupled with the inherent breathability and flexibility afforded by its nonwoven architecture, these integrated characteristics ensure reliable operational stability and wearing comfort for practical flexible thermoelectric devices. To further assess the durability of the fabric under practical conditions, we evaluated its mechanical and environmental stability. The fabric exhibited outstanding bending durability, as evidenced by negligible change in electrical resistance after 5000 bending cycles (Fig. S15). Environmental stability tests revealed that after 14 days of storage under ambient conditions (18–23 °C, 55–68% RH), the power factor retained approximately 95% of its initial value (Fig. S16). Even under high humidity (80% RH) for 7 days, the power factor remained at 94% of the original (Fig. S17). These results collectively confirm the robust operational reliability of the PEDOT:PSS nonwoven fabric for practical wearable applications. To evaluate the potential of PEDOT:PSS fabrics for wearable thermoelectric applications, we fabricated flexible thermoelectric devices using optimally processed samples that underwent H_2_SO_4_-NaOH post-treatment. The flexible device consists of twenty-four thermoelectric legs fabricated from PEDOT:PSS fabric (40 mm × 8 mm), connected in series using silver paste and copper wires (Fig. 4g). The open-circuit voltage increased linearly with the temperature difference (Δ*T*), rising from 3.6 mV at Δ*T* = 5 K to 23.1 mV at Δ*T* = 30 K (Fig. 4h). The relationship between open-circuit voltage, output power, and current at different Δ*T* (Δ*T* = 8, 15, and 30 K) is shown in Fig. 4i. At a Δ*T* of 30 K, the maximum output power was approximately 242 nW, corresponding to an area-normalized power density of 31.5 μW m^−2^. While this power level is modest, it serves as a proof-of-concept for our material and device architecture. Based on the current power density of 31.5 μW m^−2^, achieving an output power of 1 μW would require an active area of approximately 0.032 m^2^. These estimates highlight that further improvements in power density are necessary for practical wearable applications. The reliability of the device design was further verified using a twenty-leg configuration (Fig. S18), demonstrating consistent performance trends. Furthermore, to demonstrate the versatility of our microfluidic-spun microfibers beyond thermoelectric applications, we explored their potential in microscale energy storage. As shown in Fig. S19, three parallel fiber-based supercapacitors were successfully integrated within a compact area, yielding a specific capacitance of 48.1 F g^−1^. This result confirms the feasibility of constructing high-density micro-supercapacitors from our microfibers.

### Design and Application of Radiation-Modulated Planar Fabric

**Fig. 5:**
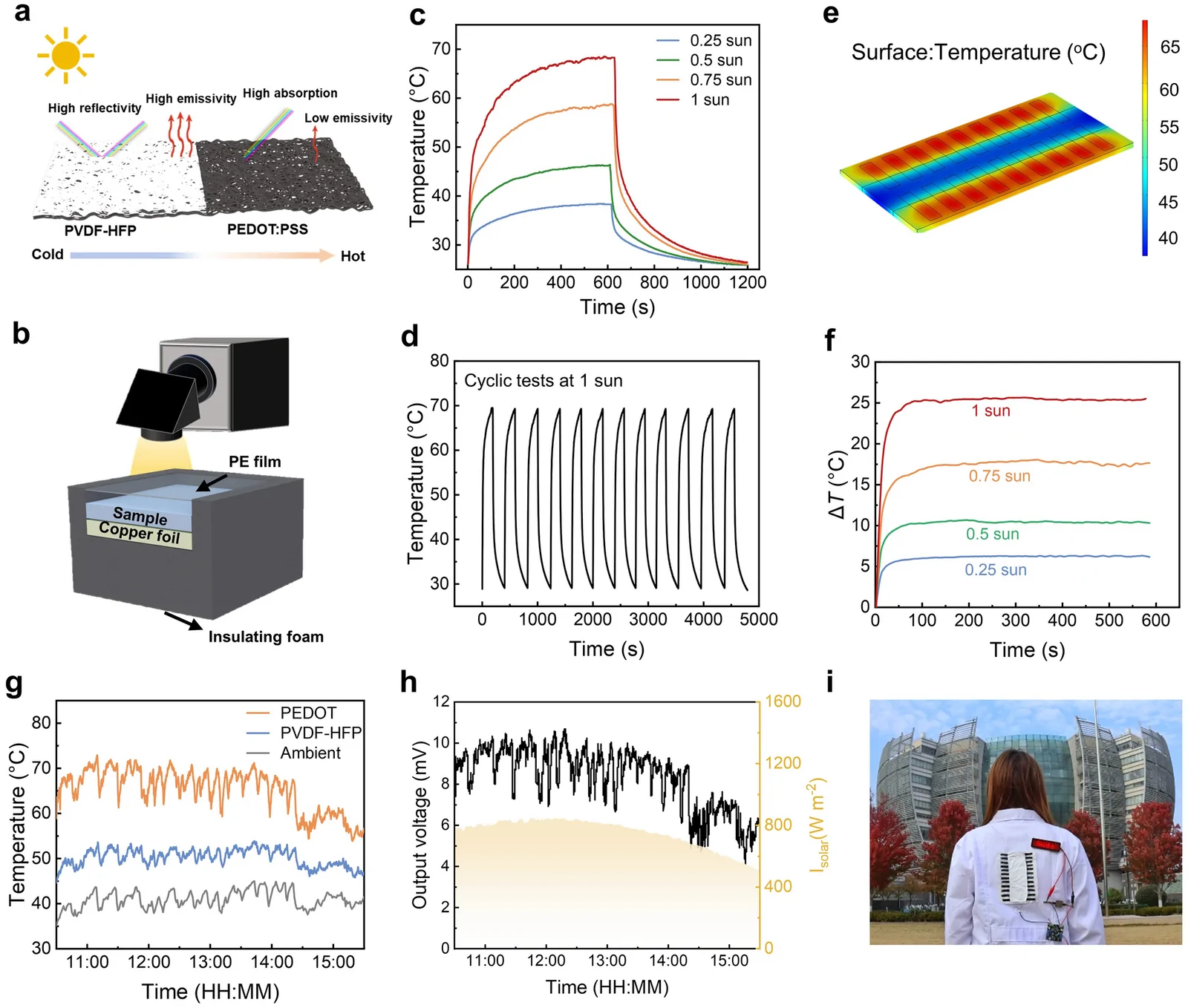
Design and application of radiation-modulated planar fabric. **a** Principle and structure of the radiation-modulated planar fabric. **b** Schematic diagram of the indoor photothermal test device. **c** Temperature rise of the PEDOT:PSS fabric under simulated solar irradiance from 0.25 to 1 sun. **d** Cyclic heating–cooling stability test of the PEDOT:PSS fabric over 12 cycles at 1 sun. **e** Simulated temperature distribution across the radiation-modulated planar fabric with 24 pairs under 1 sun illumination (finite element analysis). **f** Measured temperature difference (Δ*T*) of the radiation-modulated planar fabric under varying irradiance (0.25–1 sun). **g** Comparative temperatures of the ambient environment, PVDF-HFP nanofiber membrane, and PEDOT:PSS fabric during outdoor testing. **h** Real-time output voltage of the fabric under natural sunlight. **i** Photograph of the thermoelectric device powering an LED screen

By integrating the fabricated PEDOT:PSS nonwoven fabric with an electrospun poly(vinylidene fluoride-co-hexafluoropropylene) (PVDF-HFP) membrane, we constructed a radiation-modulated planar fabric (Fig. 5a). The operational principle leverages a combination of photothermal conversion and passive radiative cooling to create a sustained in-plane temperature gradient. The PEDOT:PSS functions as a photothermal unit that absorbs solar radiation and converts it into heat, while the adjacent PVDF-HFP region acts as a radiative cooler, dissipating thermal energy via mid-infrared emission and solar reflection. This design transforms incoming solar energy into a spatially nonuniform temperature field, which establishes a temperature difference (Δ*T*) across the device surface to drive thermoelectric power generation. Effective radiative cooling is enabled by the PVDF-HFP fiber membrane, which exhibits an average solar reflectance of 93.7% and an average mid‑infrared emissivity of 88%, whereas the PEDOT:PSS fabric exhibits excellent photothermal conversion owing to its high average solar absorptance of 89% and an average mid‑infrared emissivity of 32% (Fig. S20) [59]. The photothermal performance of the PEDOT:PSS fabric was characterized using an indoor photothermal test device (Fig. 5b). Under simulated solar irradiation intensities of 0.25, 0.5, 0.75, and 1 sun, the fabric surface temperature increased to 38.1, 46.3, 58.8, and 68.3 °C, respectively (Fig. 5c). This irradiance-dependent temperature response, together with the strong positive correlation between temperature rise and light intensity (Fig. S21), confirms the material’s stable and controllable photothermal behavior. Durability was assessed over 12 consecutive heating–cooling cycles, with the maximum temperature remaining stable at approximately 69.3 °C in response to 1 sun illumination and showing minor fluctuation (Fig. 5d), indicating reliable cyclic endurance. Under 1 sun illumination, the substrate layer gradually warmed to 52.7 °C after 1300 s, while the radiative-cooling layer reached only 43.9 °C under the same conditions (Fig. S22). This confirms the passive radiative-cooling capability of the PVDF-HFP membrane, which is essential for establishing the in-plane temperature difference. Finite element simulations were conducted under standard conditions (25 °C ambient temperature, 1 sun illumination) to predict the temperature gradient, yielding a stable in-plane Δ*T* of approximately 28 °C, with the hot side at 66 °C and the cold side at 38 °C (Fig. 5e). Experimental measurements corroborated this trend, showing Δ*T* increase from 6.3 to 25.4 °C as irradiance rose from 0.25 to 1 sun (Fig. 5f). This temperature gradient results from a heat flux imbalance, where the PEDOT:PSS side absorbs ~ 800–900 W m^−2^ of solar radiation compared to only ~ 50–100 W m^−2^ on the PVDF-HFP side, creating a net heat flux difference of ~ 200–300 W m^−2^ that drives in-plane thermal conduction. The close agreement confirms the fabric’s ability to establish an effective temperature gradient across varying light intensities. To validate the performance in real environments, outdoor testing was conducted on August 22 in Suzhou, China (31° 18′ 6.1″ N, 120° 34′ 51.9″ E), under clear sky conditions. Schematic and physical diagrams of the testing apparatus are shown in Figs. S23 and S24. To ensure accuracy, thermocouples were placed between samples and foam substrates, while aluminum foil-covered polystyrene foam and transparent polyethylene covers minimized radiative and convective losses. Environmental parameters recorded throughout testing are presented in Fig. S25. A detailed analysis of these environmental parameters reveals their substantial impact on radiative-cooling efficiency, which in turn affects the in-plane temperature gradient. Wind speed strongly influences convective heat transfer between the fabric and the ambient air. For radiative-cooling materials designed to achieve sub‑ambient temperatures, wind‑induced convective heating should be minimized, as it brings environmental heat to the cooler surface [60]. Ambient humidity also plays a critical role. High humidity weakens radiative-cooling efficiency by enhancing downward atmospheric radiation and reducing the transparency of the atmospheric window [61], which directly compromises the cooling power of the PVDF‑HFP membrane. Despite these environmental fluctuations, under natural sunlight, a pronounced temperature difference developed between the hot and cold sides of the radiation-modulated planar fabric (Fig. 5g). Real-time voltage output correlated strongly with solar irradiance, reaching a maximum of 10.7 mV at the peak solar intensity of 837 W m^−2^ (Fig. 5h), confirming effective thermoelectric energy harvesting in practical conditions. Figure S26 further shows the voltage generated during outdoor testing from 15:30 to 19:30. To explore its potential for practical wearable applications, we integrated the radiation-modulated planar fabric into clothing and captured an outdoor infrared thermal image (Fig. S27). Furthermore, the integrated 24-pair fabric connected to a voltage amplifier successfully harvested solar energy to power an LED display screen (Figs. 5i and S28), demonstrating its capability to directly drive wearable microelectronic devices. Interfacial adhesion tests revealed an initial peel strength of 0.2 N cm^−1^ between the PVDF-HFP and PEDOT:PSS layers, which remained at approximately 95% after 7-day ambient storage and 88% after 500 bending cycles, confirming reliable bonding durability for wearable applications (Fig. S29).

## Conclusion

In summary, this work demonstrates high-performance thermoelectric nonwoven fabrics based on microfluidic-spun PEDOT:PSS microfibers, possessing tailored micro-/nanoscale physical and electronic structures. Specifically, microfluidic multichannel fluidic control combining with in situ rapid coagulation enables continuous spinning of PEDOT:PSS fibers with polymer chains orientated along the fiber axis. Subsequent acid treatment removes excess PSS and induces a coil-to-linear conformational transition of PEDOT chains. This multiscale structural ordering creates highly efficient charge-transport pathways, yielding a maximum electrical conductivity of 2857 S cm^−1^. Further fine-tuning of the Fermi level via NaOH-mediated dedoping promotes energy-dependent carrier scattering, thereby enhancing the Seebeck coefficient. This integrated strategy successfully optimizes the conventional trade-off between electrical conductivity and the Seebeck coefficient, achieving a high power factor of 179.8 μW m^−1^ K^−2^ (*σ* = 2038 S cm^−1^, *S* = 29.7 μV K^−1^), surpassing most reported organic thermoelectric fibers. Furthermore, by integrating the PEDOT:PSS fiber-based nonwoven fabric with an electrospun PVDF-HFP radiative-cooling layer, we construct a novel radiation-modulated planar fabric for direct solar thermoelectric power generation. The simultaneous photothermal conversion and passive radiative cooling on this device can establish a stable in-plane temperature gradient (Δ*T* ≈ 20 K) under natural sunlight (0.84 sun) for continuous thermoelectric energy conversion. This work establishes a versatile microfluidic spinning platform for producing high‑performance organic thermoelectric fibers/fabrics, offering a promising pathway toward self‑powered wearable electronics.

## Supplementary Information

Below is the link to the electronic supplementary material.

Supplementary file1 (DOCX 14168 KB)
