# Supplementary material for: Microfluidic Spinning Boosting Thermoelectric Performance of PEDOT:PSS Nonwoven Fabrics
Source: Nanomicro Lett. 2026 May 20;18:376. doi: 10.1007/s40820-026-02227-3 (PMC13190984; doi:10.1007/s40820-026-02227-3)
Supplement: Supplementary file 1 — Supplementary file1 (DOCX 14168 KB) [file 40820_2026_2227_MOESM1_ESM.docx]

Supporting Information for

**Microfluidic Spinning Boosting Thermoelectric Performance of PEDOT:PSS** **Nonwoven Fabrics**

Yuhui Zhang^1‡^, Hui Qiu^1‡^*, Jian Yang^1^, Pengle Cao^1^, Yu Wang^1^, An-Quan Xie^1^, Ke-Qin Zhang^1^*, Xiao-Qiao Wang^1^*

National Engineering Laboratory for Modern Silk, College of Textile and Clothing Engineering, Soochow University, Suzhou, 215123, P. R. China

*Corresponding authors. E-mail: [hqiu@suda.edu.cn](mailto:hqiu@suda.edu.cn) (Hui Qiu); [kqzhang@suda.edu.cn](mailto:kqzhang@suda.edu.cn) (Ke-Qin Zhang); [xqwang@suda.edu.cn](mailto:xqwang@suda.edu.cn) (Xiao-Qiao Wang)

**Note S1 Calculation of average UV-vis-NIR spectral reflectivity**

The average reflectivity ${(\bar{R}}_{solar})$ in the range of 0.28-2.5 μm was obtained by

$$\begin{aligned} \bar{R}_{solar}\text{=}\frac{\int_{\text{0.}\text{2}\text{8}\text{ }\text{μm}}^{\text{2.5 }\text{μm}} \text{ρ}_{\text{solar}}\left( \text{λ} \right)\text{I}_{\text{solar}}\left( \text{λ} \right)\text{ⅆ}\text{λ}}{\int_{\text{0.}\text{2}\text{8 }\text{μm}}^{\text{2.5 }\text{μm}} \text{I}_{\text{solar}}\left( \text{λ} \right)\text{ⅆ}\text{λ}} \end{aligned}(S1)$$

where *λ* is the wavelength of the incident light, ranging from 0.3 to 2.5 μm, *ρ_solar_(λ)* is the surface spectral reflectance of samples and *I_solar_(λ)* is the normalized ASTM G173 Global solar radiation intensity spectrum.

**Note S2 Calculation of average MIR spectral emissivity**

The average emissivity ($\overline{\text{ε}}$) in the "atmospheric window" (8-13 μm) can be calculated by:

$$\overline{\text{ε}}\text{=}\frac{\int_{\text{8 }\text{μm}}^{\text{13 }\text{μm}} \text{ε}\left( \text{λ}\text{,}\text{ }\text{T} \right)\text{I}_{\text{BB}}\left( \text{λ}\text{,}\text{ }\text{T} \right)\text{ⅆ}\text{λ}}{\int_{\text{8 }\text{μm}}^{\text{13 }\text{μm}} \text{I}_{\text{BB}}\left( \text{λ}\text{,}\text{ }\text{T} \right)\text{ⅆ}\text{λ}} (S2)$$

where *I_BB_* is the spectral intensity of blackbody emission, and *ε* (*λ*, *T*) the emittance of the sample at a given temperature *T* and wavelength *λ*. In general, *T* = 300 K, *λ* in the band of 8-13 µm.

**Note S3 Finite element simulation**

The finite element simulation was performed using COMSOL Multiphysics by coupling the Heat Transfer in Solids module and the Surface-to-Surface Radiation module. The simulation was conducted under steady-state conditions. The model geometry, with a total area of 10 cm × 5 cm, consists of two adjacent regions representing the PEDOT:PSS photothermal layer and the PVDF-HFP radiative cooling layer. Solar irradiance of 1000 W m^-2^ (1sun) was applied as an incident heat flux on the top surface. An ambient temperature of 25 °C was set as the external boundary condition. The material properties were defined as follows: thermal conductivity of 0.35 W m^-1^ K^-1^ for the PEDOT:PSS layer and 0.20 W m^-1^ K^-1^ for the PVDF-HFP layer. The PEDOT:PSS photothermal layer was assigned a solar reflectance of 10% and an mid-infrared emissivity of 35%, whereas the PVDF-HFP radiative cooling layer was assigned a solar reflectance of 95% and an mid-infrared emissivity of 90%.

**Supplementary Figures and Tables**


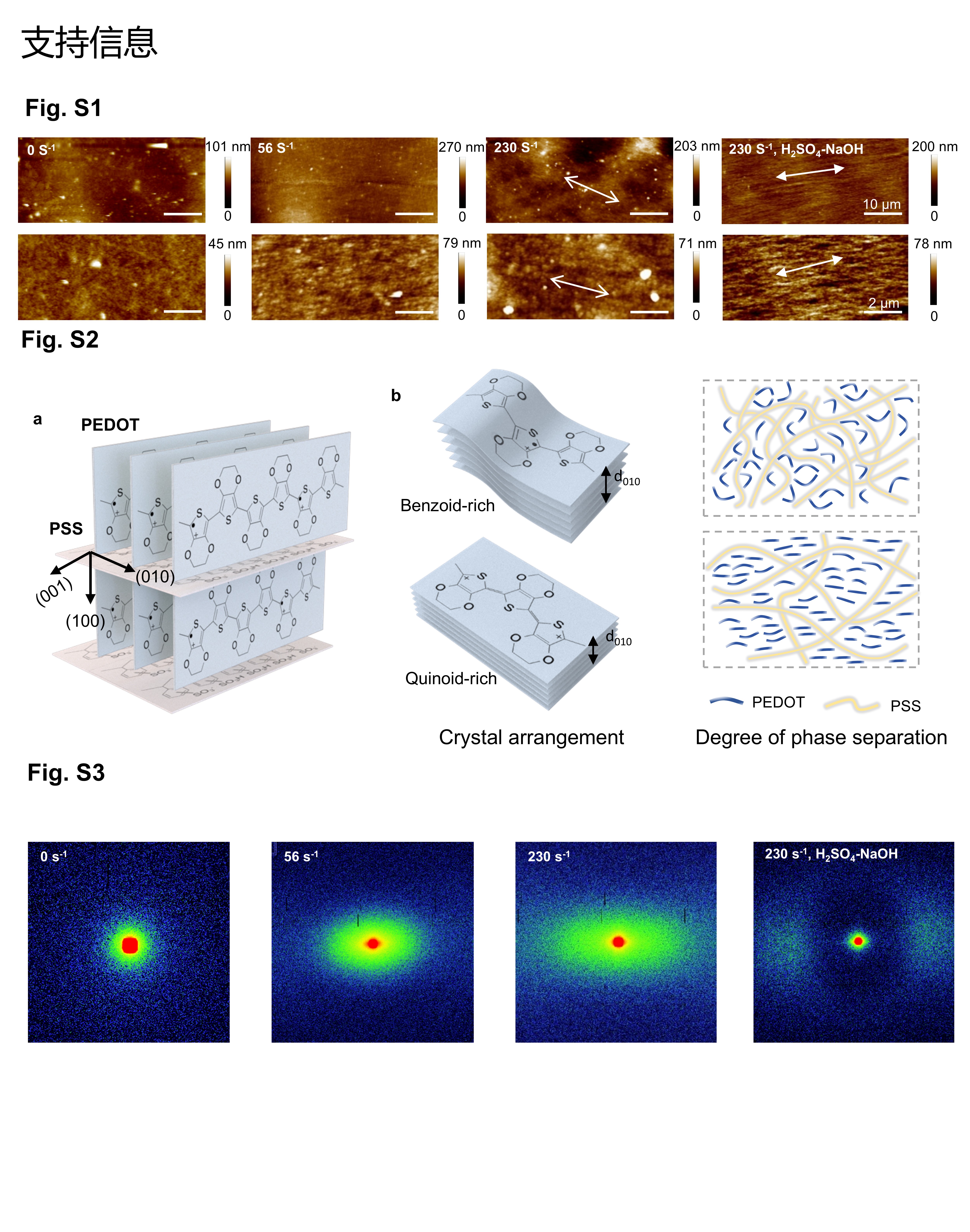


**Fig. S1** AFM images of a drop-cast film (0 s^-1^), fibers sheared at 56 s^-1^ and 230 s^-1^, and the 230 s^-1^ fiber after H_2_SO_4_-NaOH treatment.


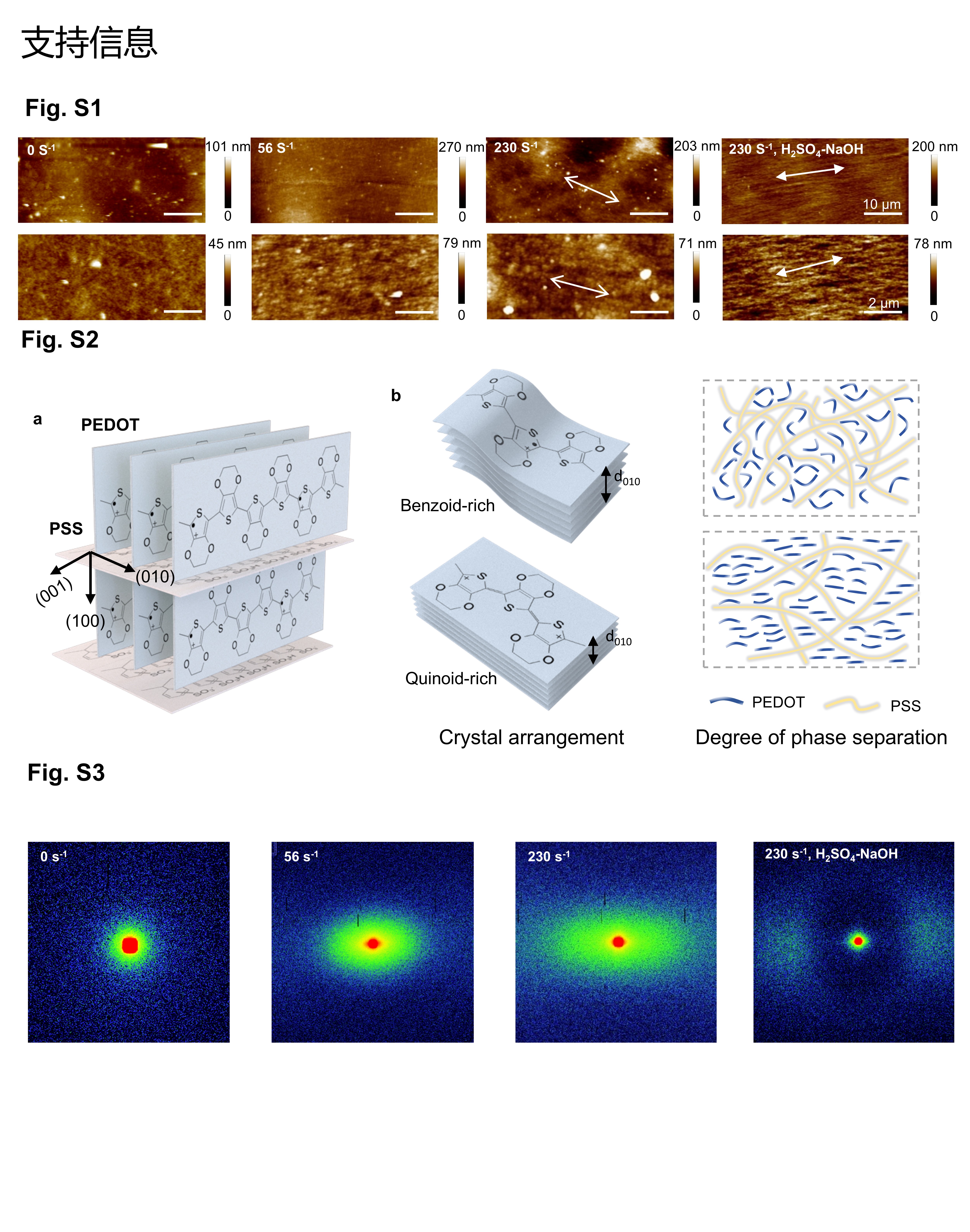


**Fig. S2 a** Schematic of the PEDOT crystal structure. **b** Schematic depicting the transformation in crystal arrangement and degree of phase separation through shear-induced orientation and H_2_SO_4_-NaOH treatment.


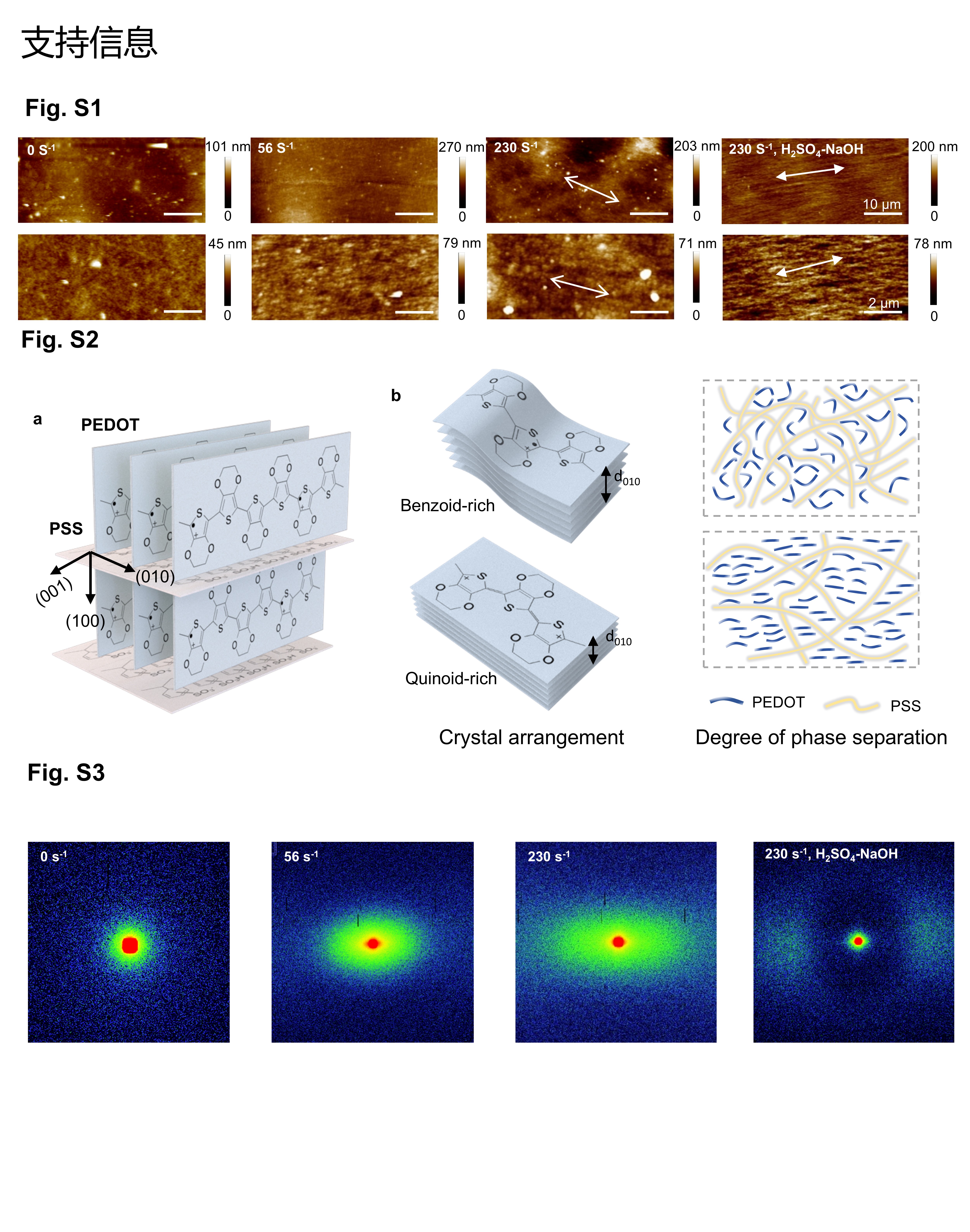


**Fig. S3** 2D-SAXS profiles of a drop-cast film (0 s^-1^), fibers sheared at 56 s^-1^ and 230 s^-1^, and the 230 s^-1^ fiber after H_2_SO_4_-NaOH treatment.


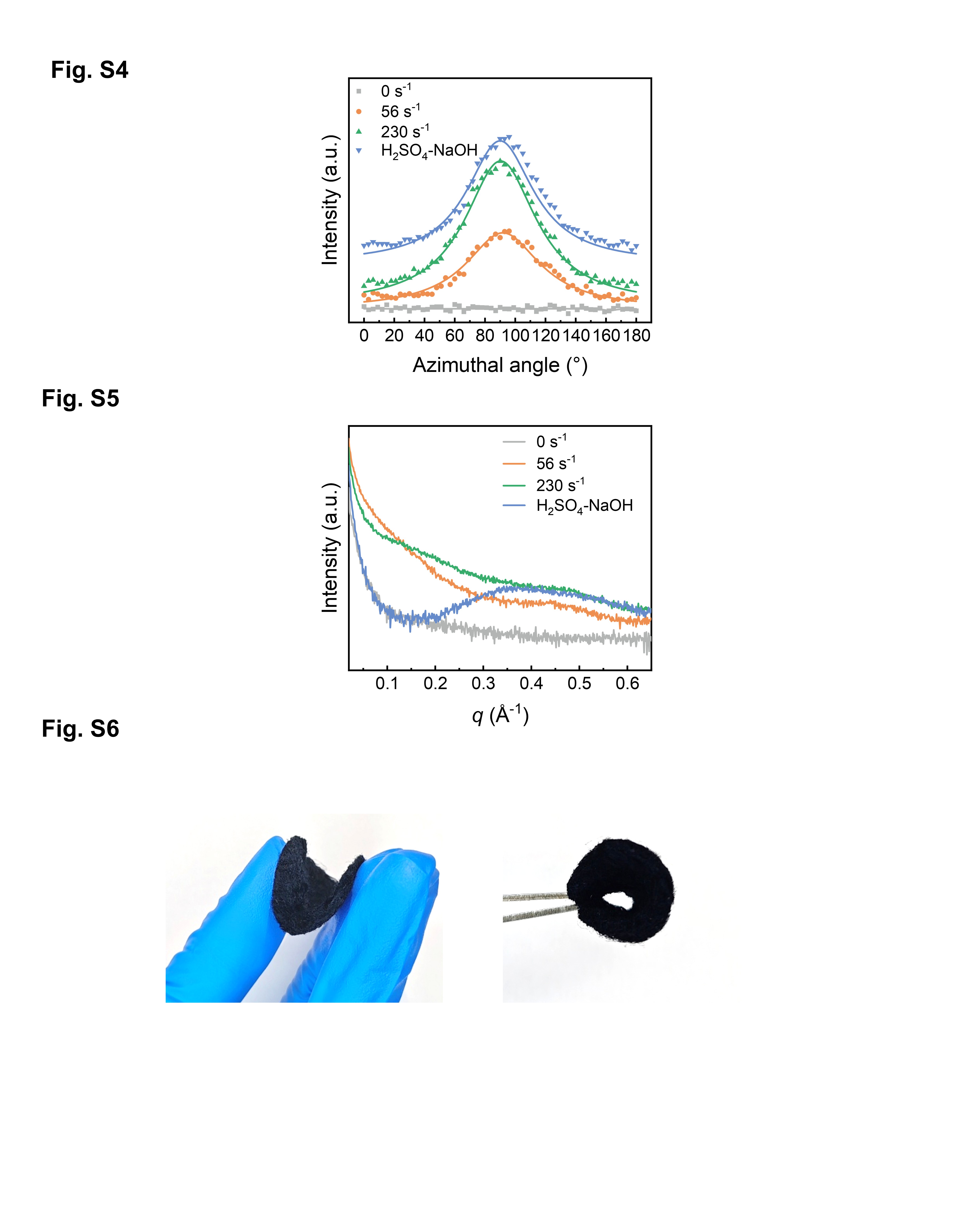


**Fig. S4** Azimuthally dependent scattering spectra of a drop-cast film (0 s^-1^), fibers sheared at 56 s^-1^ and 230 s^-1^, and the 230 s^-1^ fiber after H_2_SO_4_-NaOH treatment.


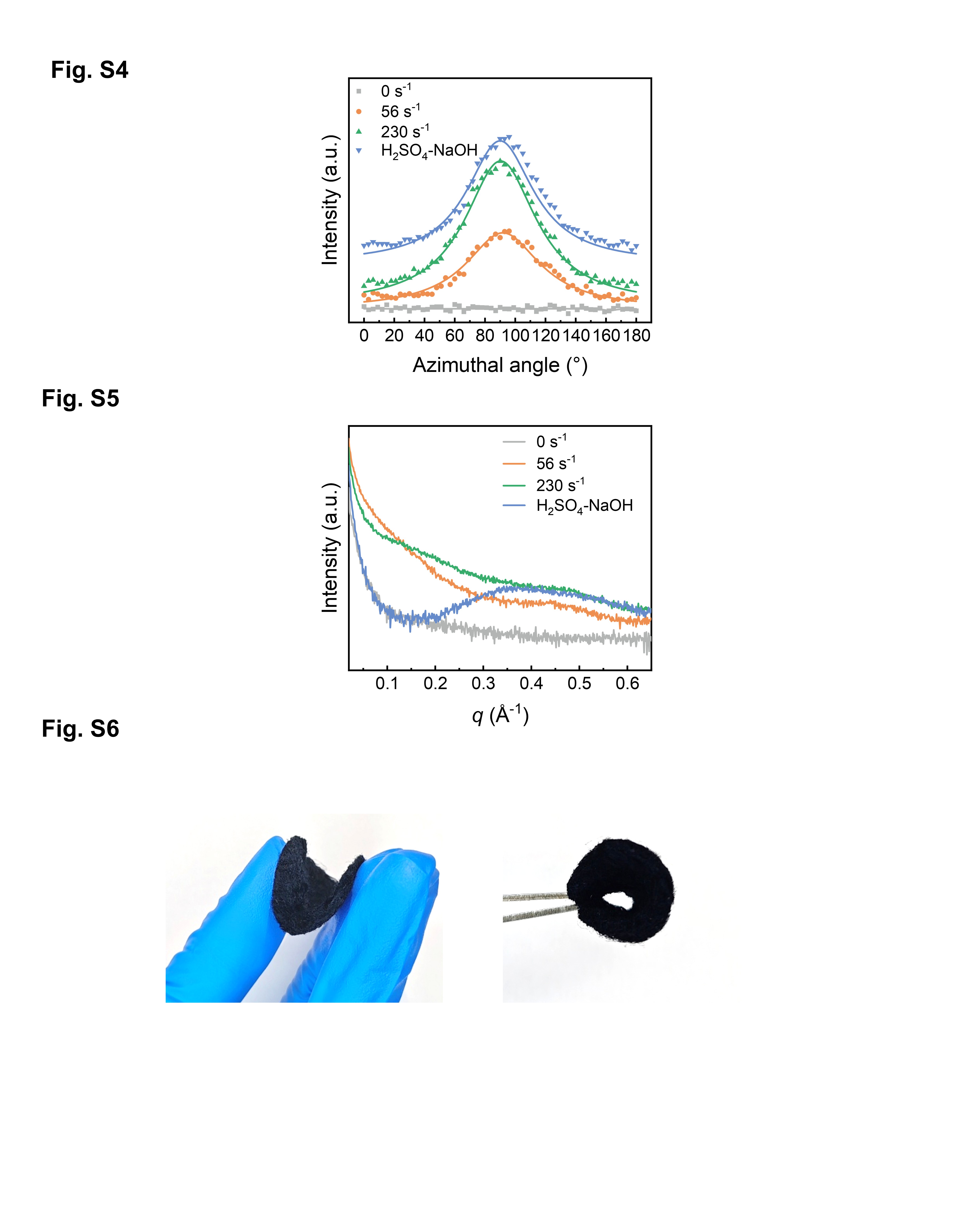


**Fig. S5** 1D SAXS profiles of a drop-cast film (0 s^-1^), fibers sheared at 56 s^-1^ and 230 s^-1^, and the 230 s^-1^ fiber after H_2_SO_4_-NaOH treatment.


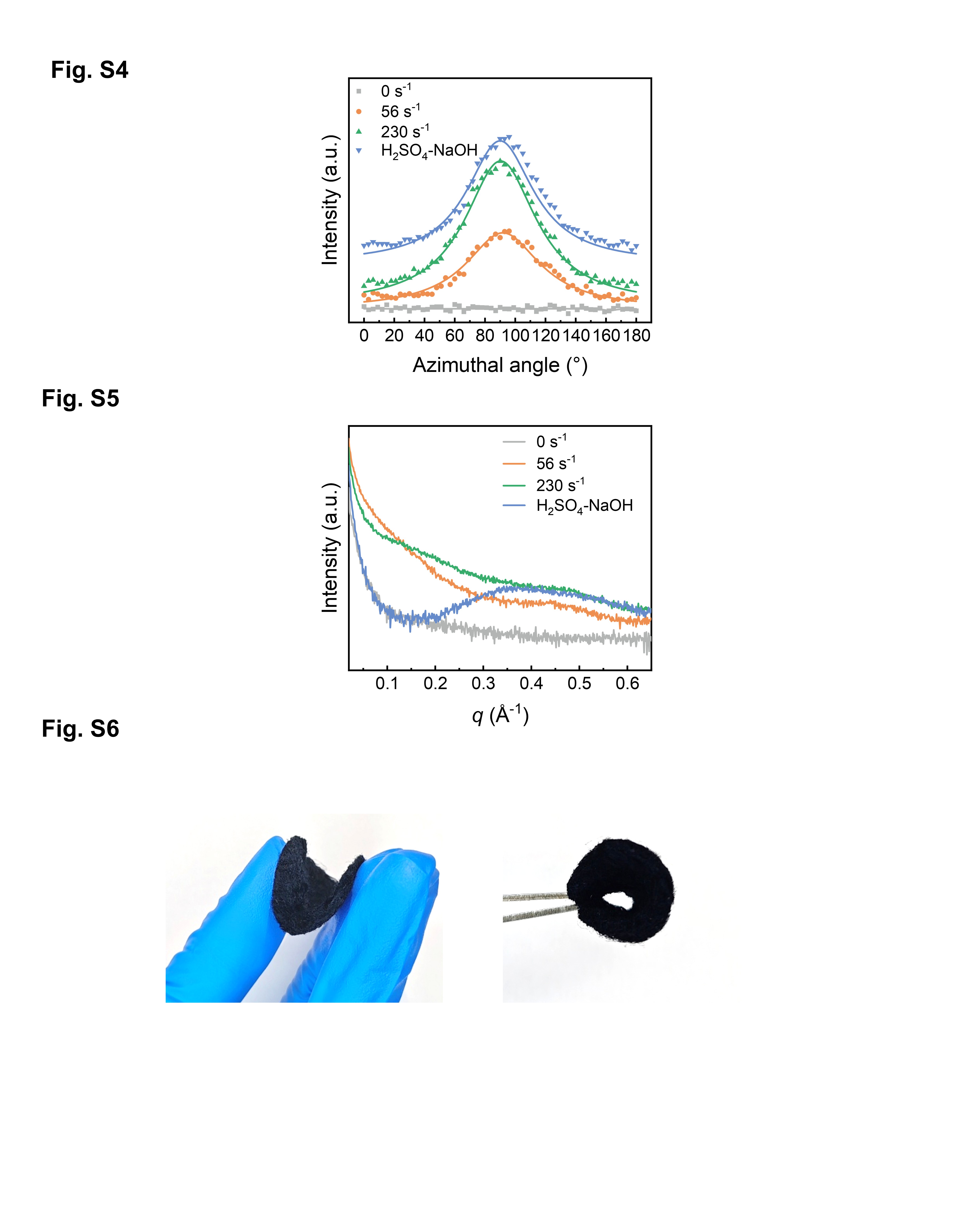


**Fig. S6** Digital photographs of the PEDOT:PSS fabric (left) and its folding state (right), demonstrating its flexibility.


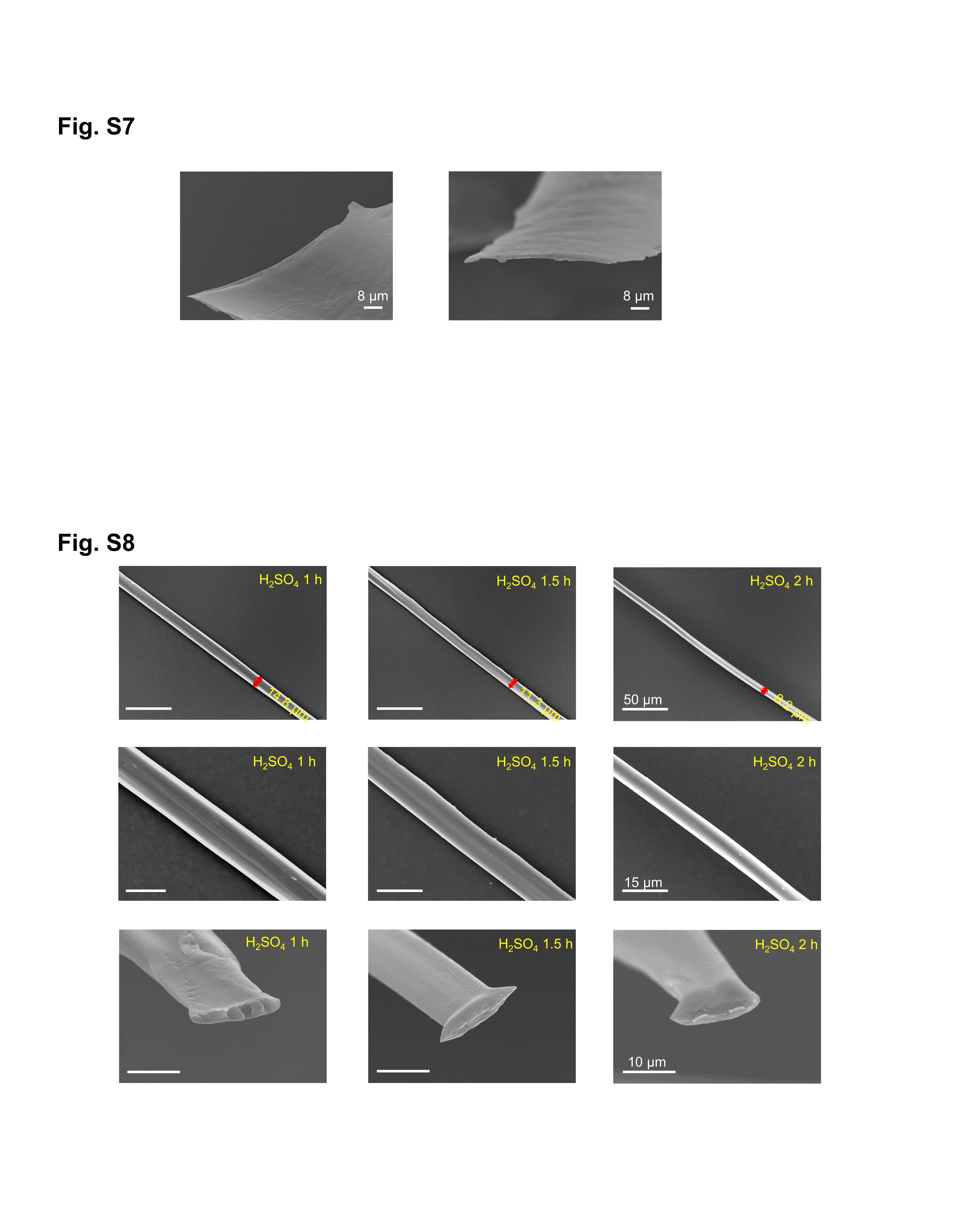


**Fig. S7** Cross-sectional SEM images of the PEDOT:PSS fibers sheared at 56 s^-1^ and 230 s^-1^.


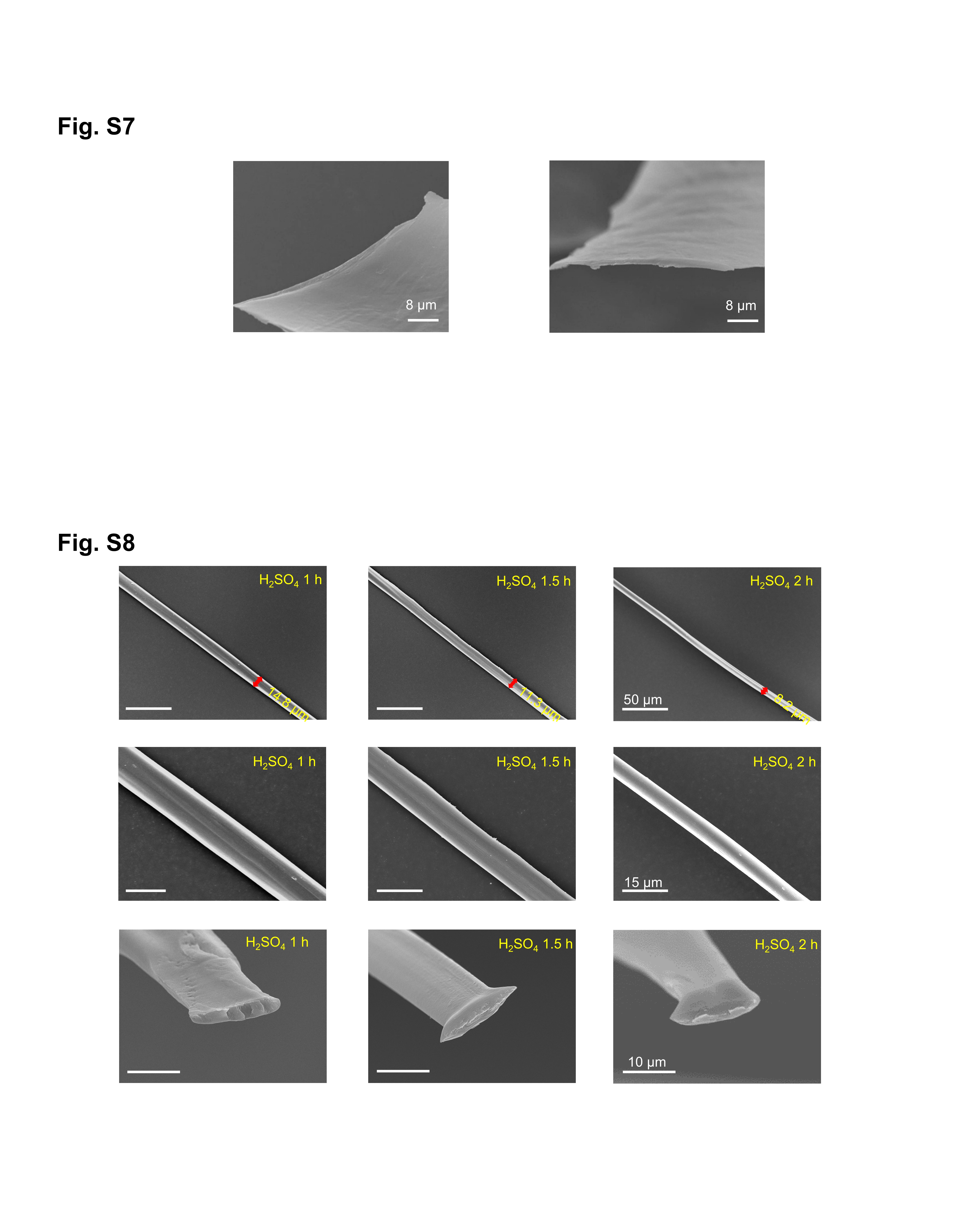


**Fig. S8** SEM images showing the plan-view and corresponding cross-sectional morphologies of the PEDOT:PSS fibers treated with H_2_SO_4_ for 1 h, 1.5 h, and 2 h.


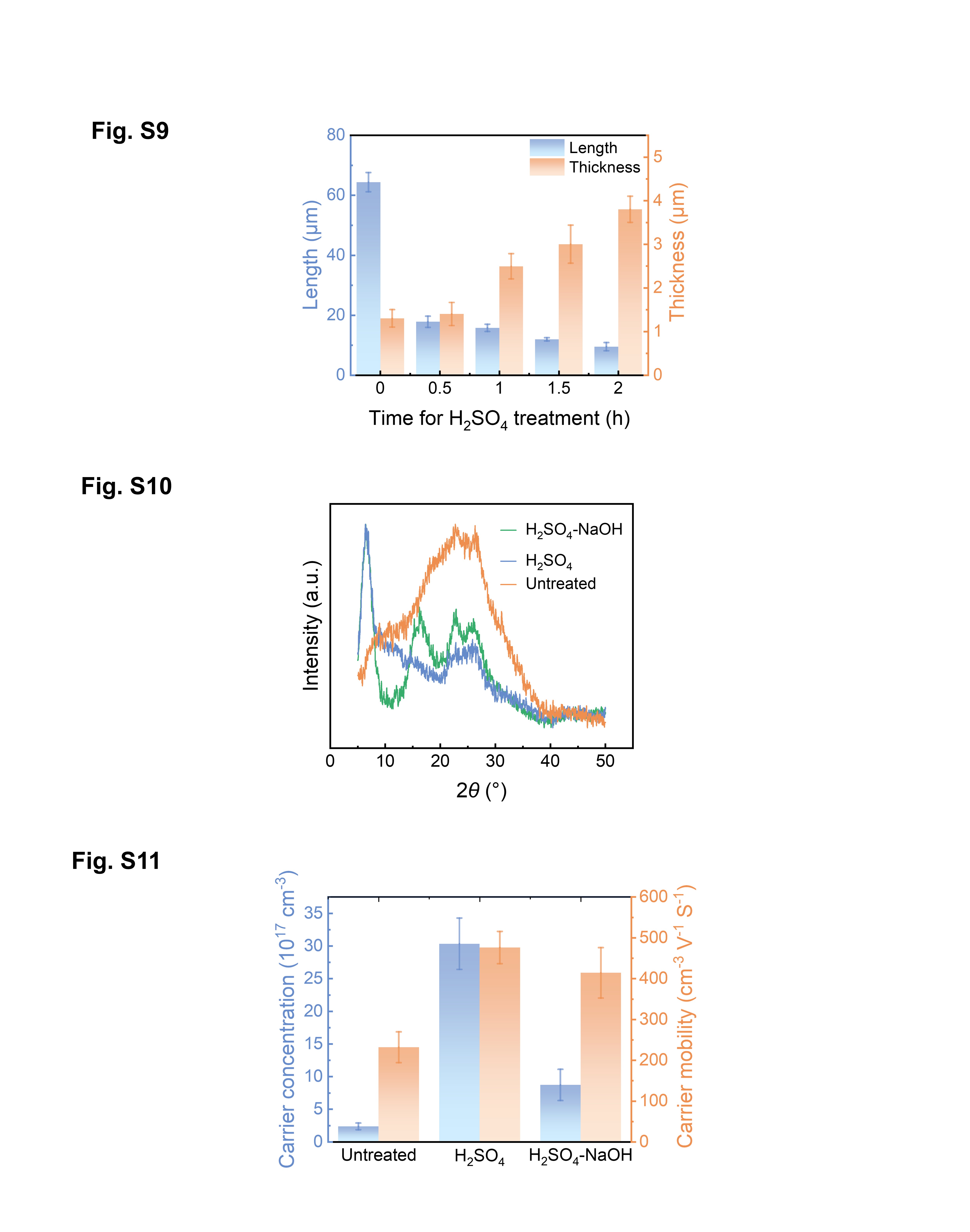


**Fig. S9** Length and thickness of the PEDOT:PSS fibers after treatment with H_2_SO_4_ for 0 h, 0.5 h, 1 h, 1.5 h and 2 h.


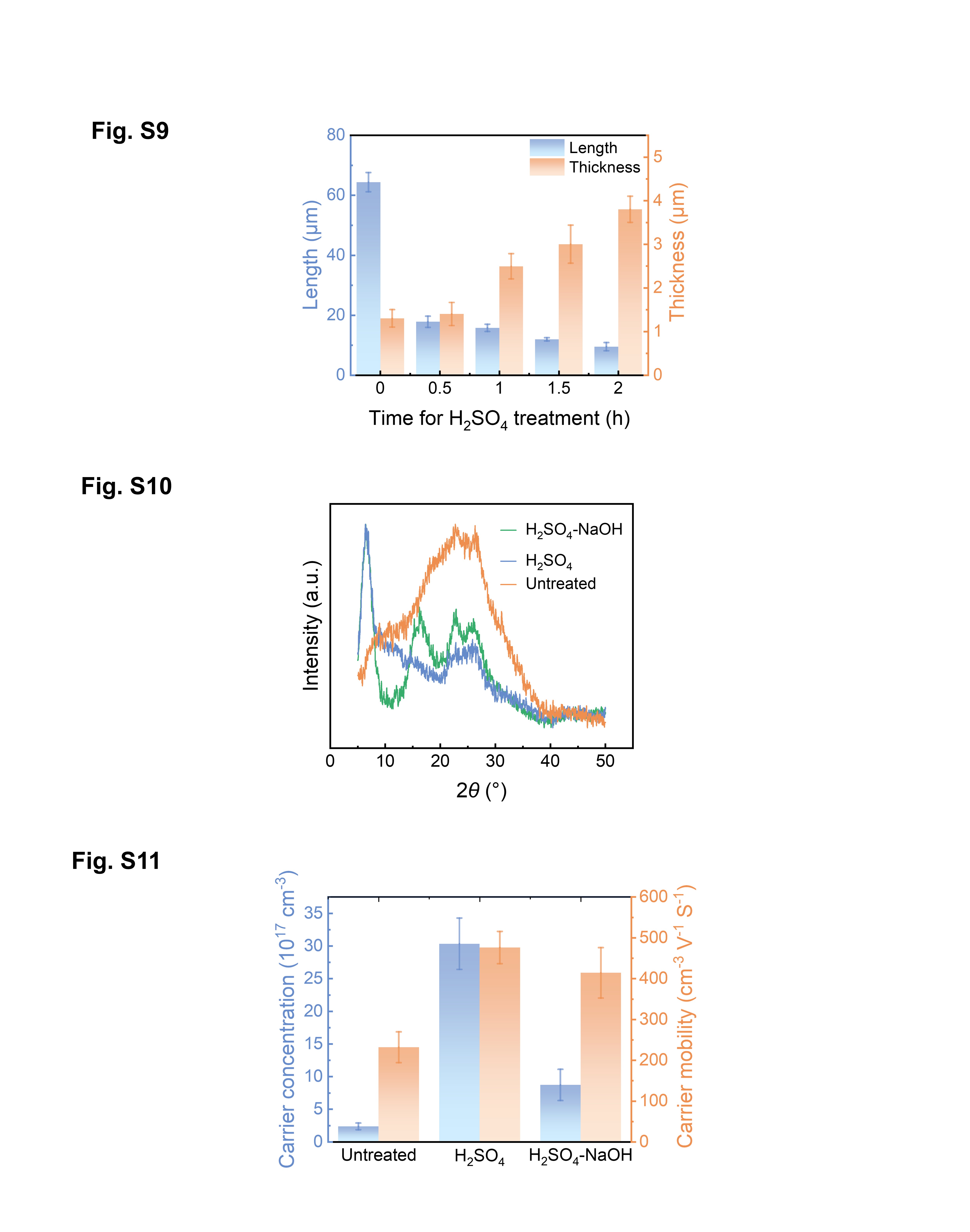


**Fig. S10** XRD patterns of the untreated, H_2_SO_4_-treated, and H_2_SO_4_-NaOH treated PEDOT:PSS fibers.

**
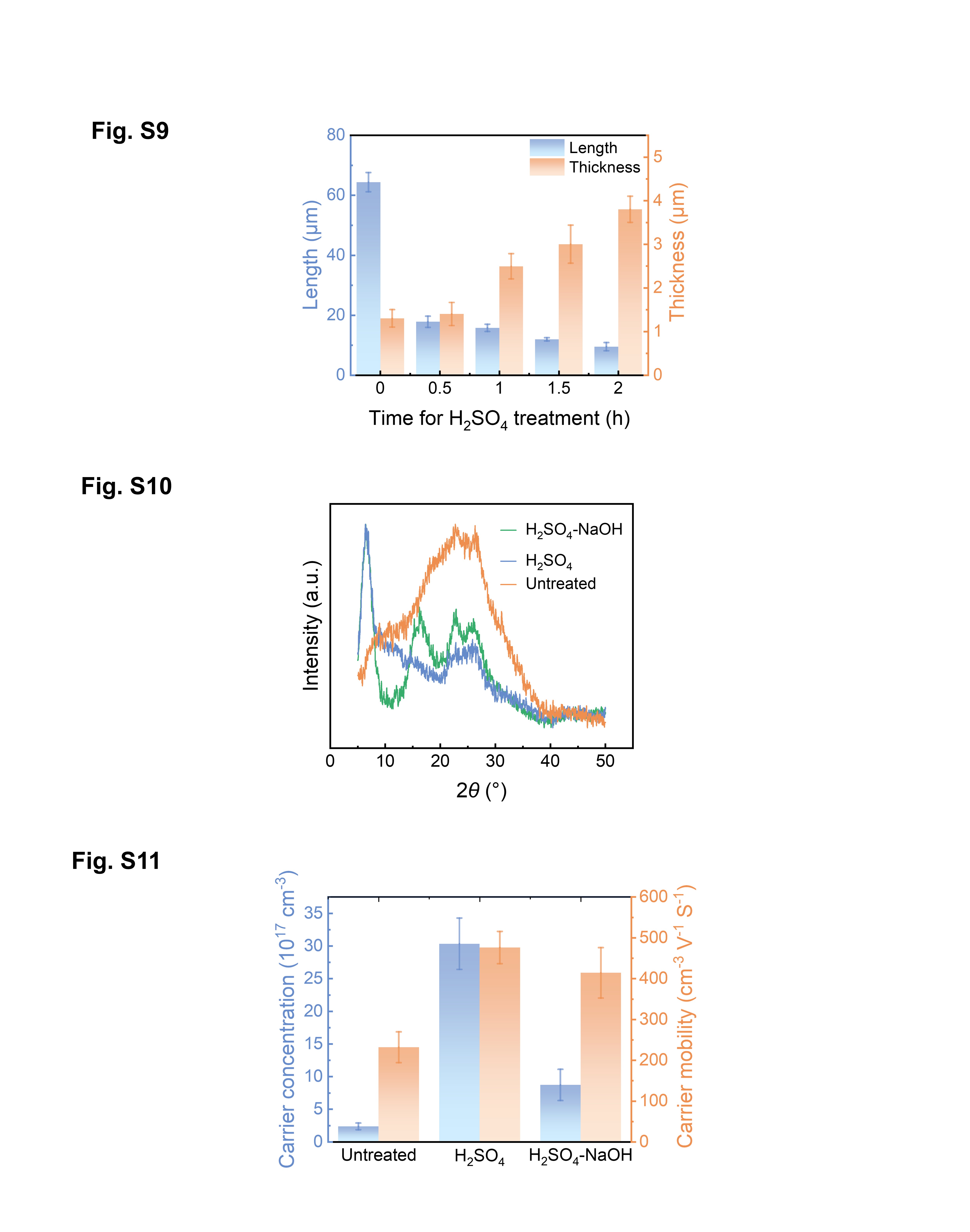
**

**Fig. S11** Carrier concentration and mobility of the untreated, H_2_SO_4_-treated, and H_2_SO_4_-NaOH treated PEDOT:PSS fibers.


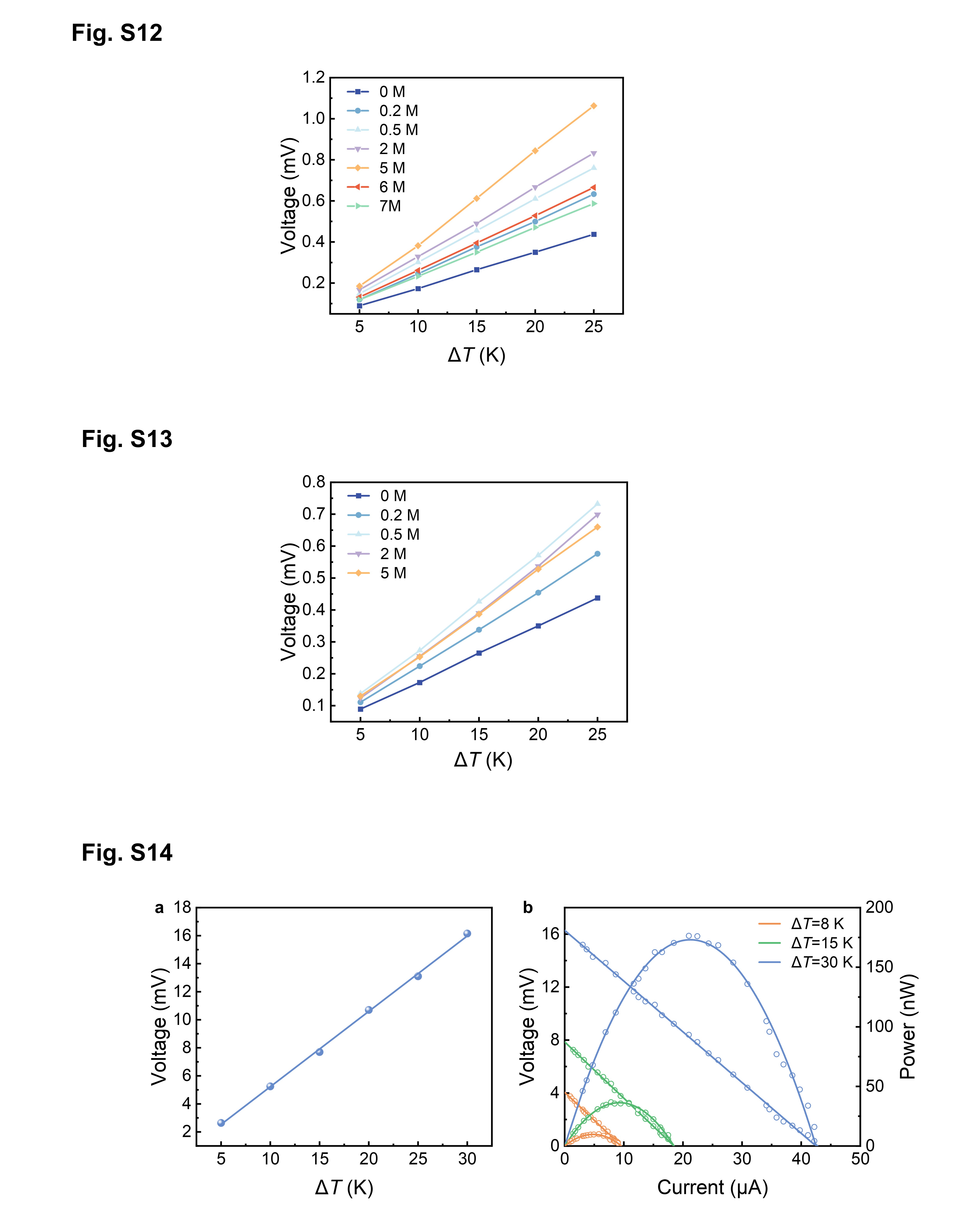


**Fig. S12** Output voltage versus applied temperature difference (Δ*T*) for the NaOH-treated PEDOT:PSS fiber.


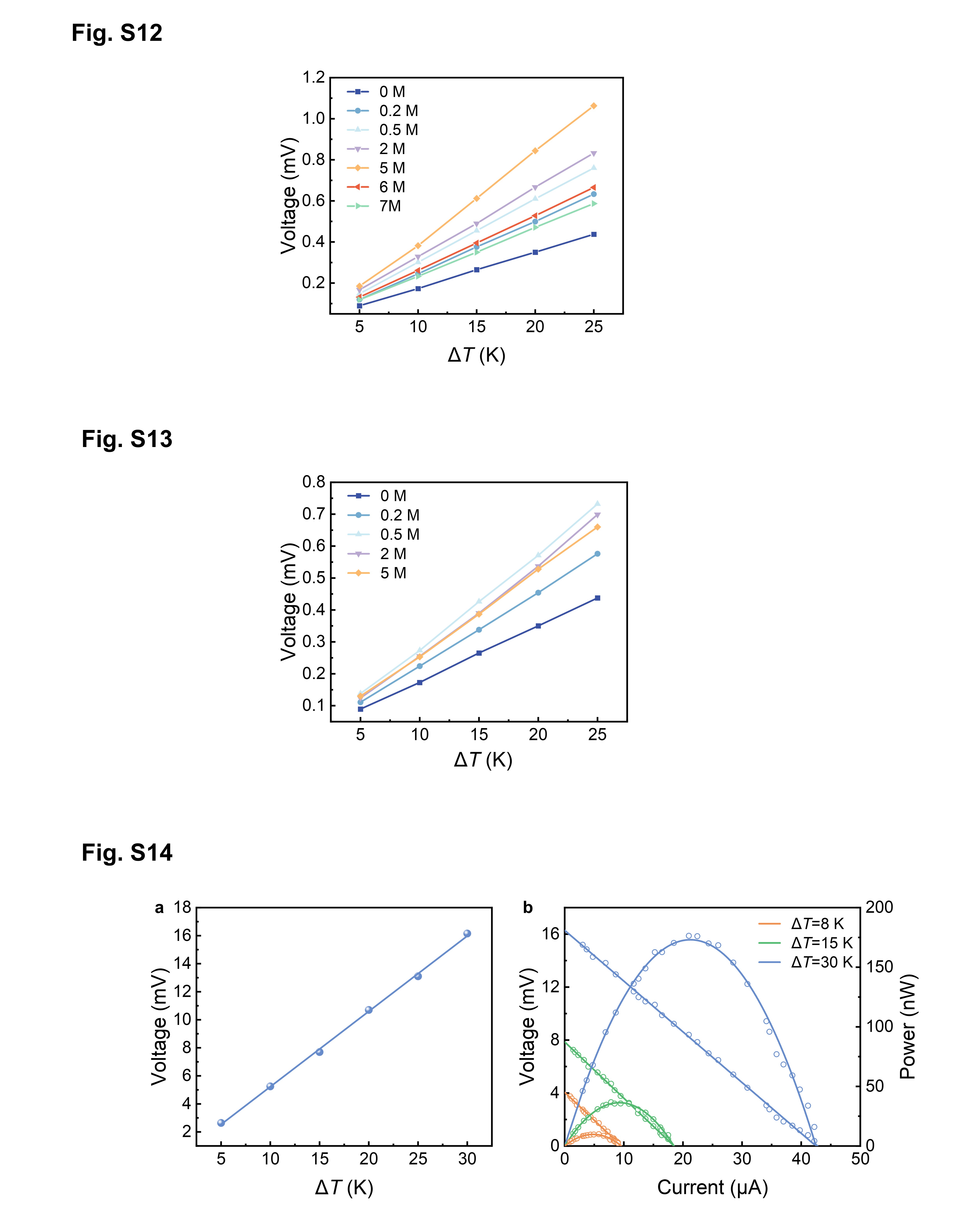


**Fig. S13** Output voltage versus temperature difference (Δ*T*) for the H_2_SO_4_-NaOH treated PEDOT:PSS fiber.

**Fig. S14** Thermoelectric performance comparison of microfluidic-spun PEDOT:PSS fiber and drop-cast film after identical H_2_SO_4_-NaOH post-treatments.


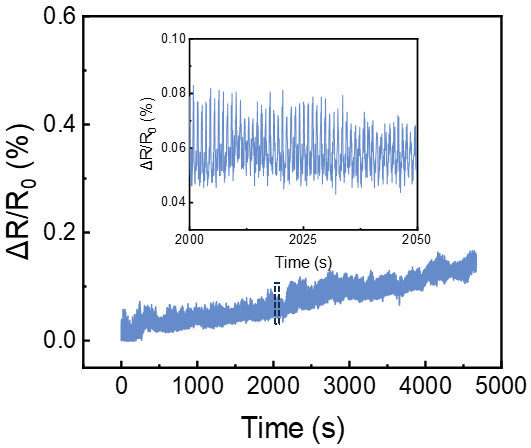


**Fig. S15** Relative resistance change of the PEDOT:PSS nonwoven fabric during 5000 bending cycles.

**Fig. S16** Thermoelectric performance of the PEDOT:PSS nonwoven fabric over long-term storage under ambient air conditions (temperature range: 18–23 °C; relative humidity: 55–68%).

**Fig. S17** Thermoelectric performance of the PEDOT:PSS nonwoven fabric under high humidity conditions (80% RH).


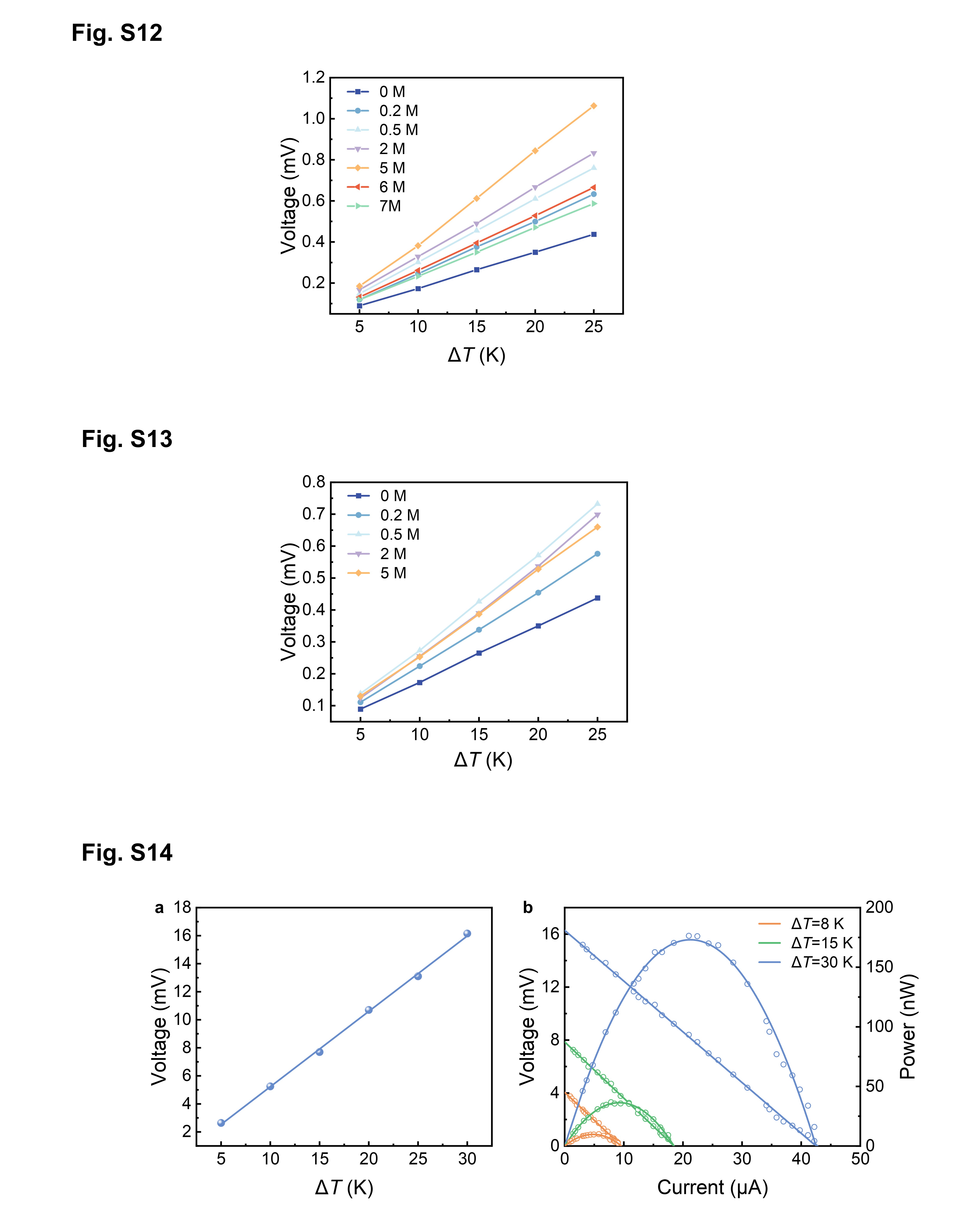


**Fig. S18 a The open-circuit voltage as a function of the temperature difference (Δ*T*). b The output voltage and output power as a function of output current at temperature differences of 8, 15, and 30 K.**

**
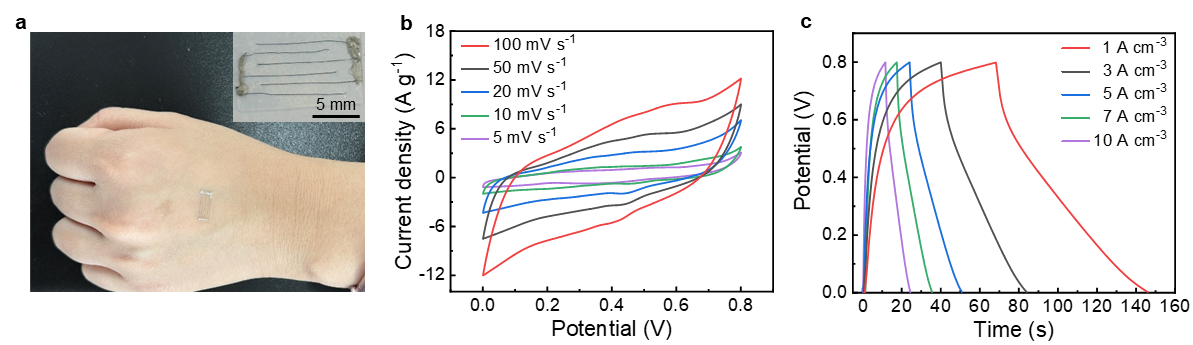
**

**Fig. S19** Electrochemical performances of micro-supercapacitors. a Photograph of the micro-supercapacitors attached to a hand. b CV curves of PEDOT:PSS fiber supercapacitors based on H_2_SO_4_/PVA. c GCD curves of PEDOT:PSS fiber supercapacitors based on H_2_SO_4_/PVA.

**
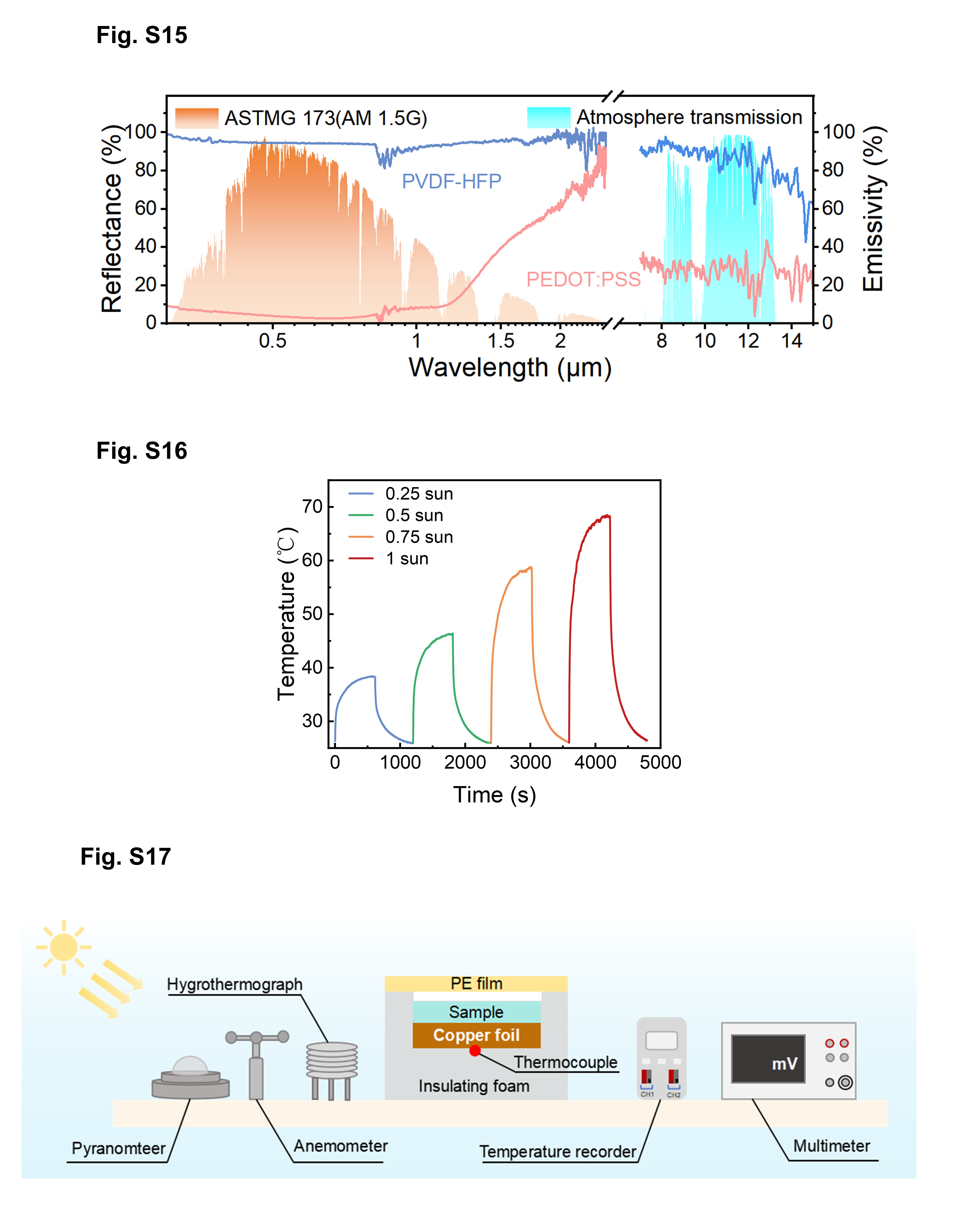
**

**Fig. S20** Reflectance and emissivity spectra of the PVDF-HFP nanofiber membrane and PEDOT:PSS fabric.

**
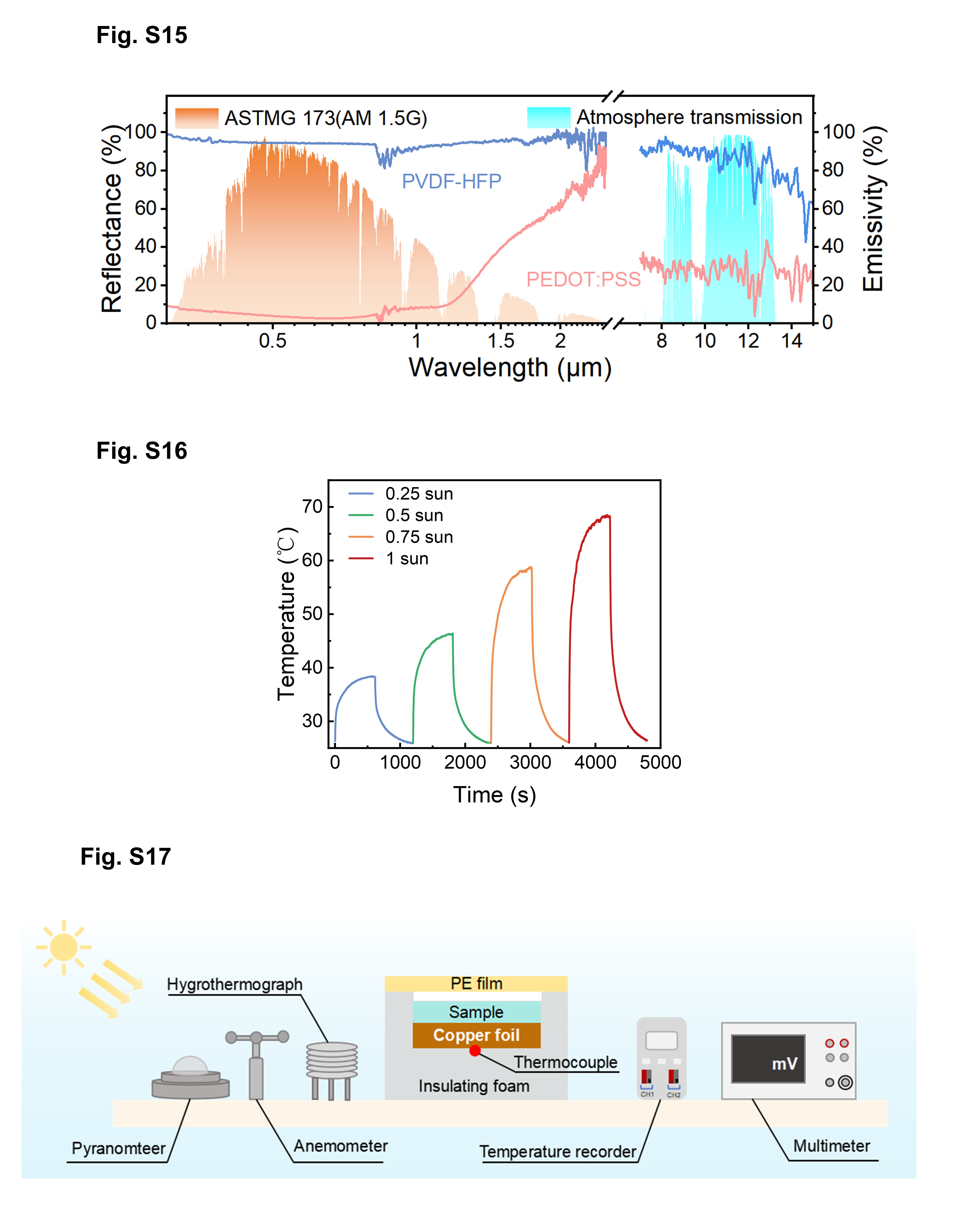
**

**Fig. S21** Temperature variations of the PEDOT:PSS fabric at 0.25, 0.5, 0.75, and 1 sun.

**Fig. S22** Temperatures of photothermal layer, substrate layer, and radiative cooling layer after a period of irradiation with 1-sun intensity.

**
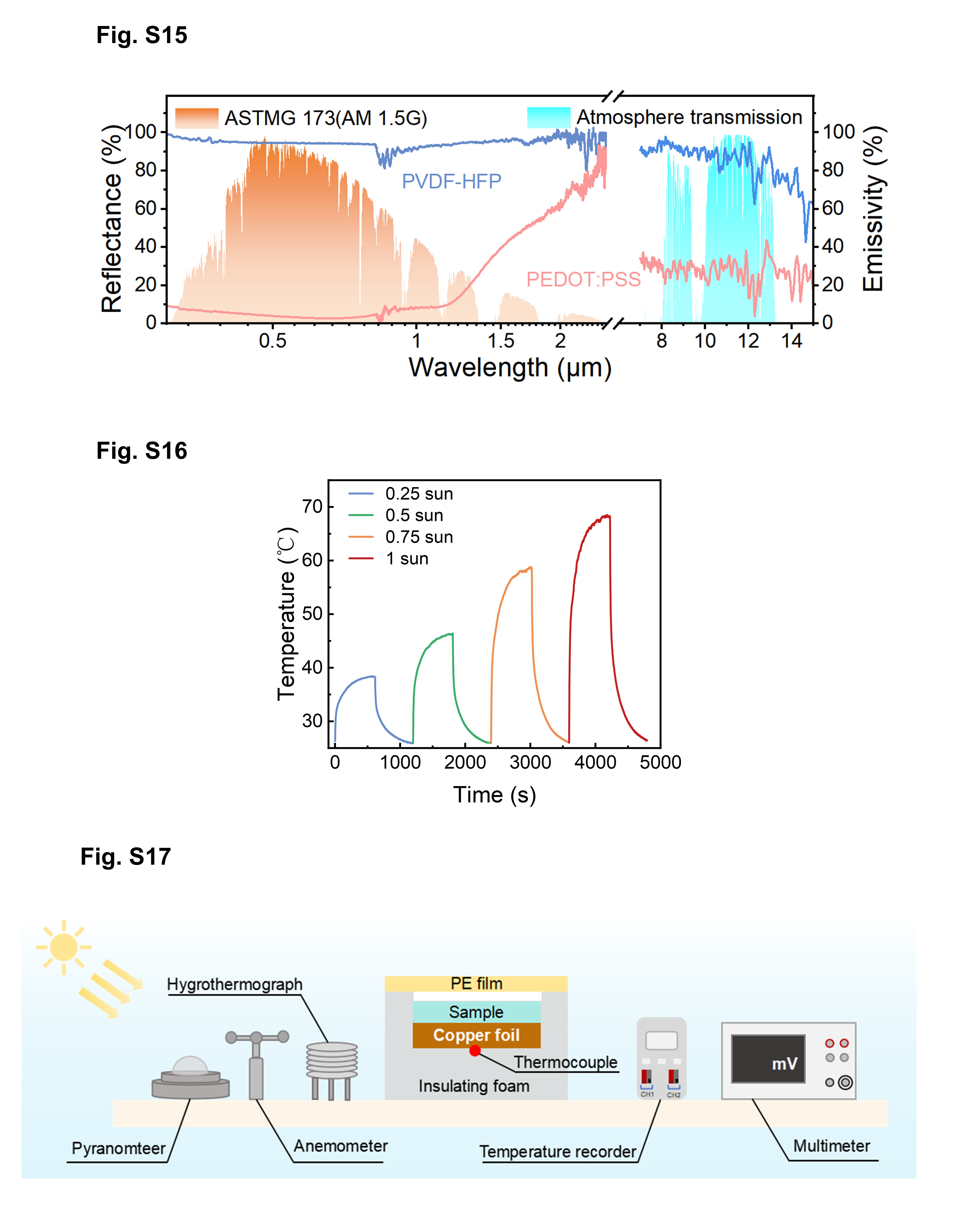
Fig. S23** Schematic diagram of outdoor test device.


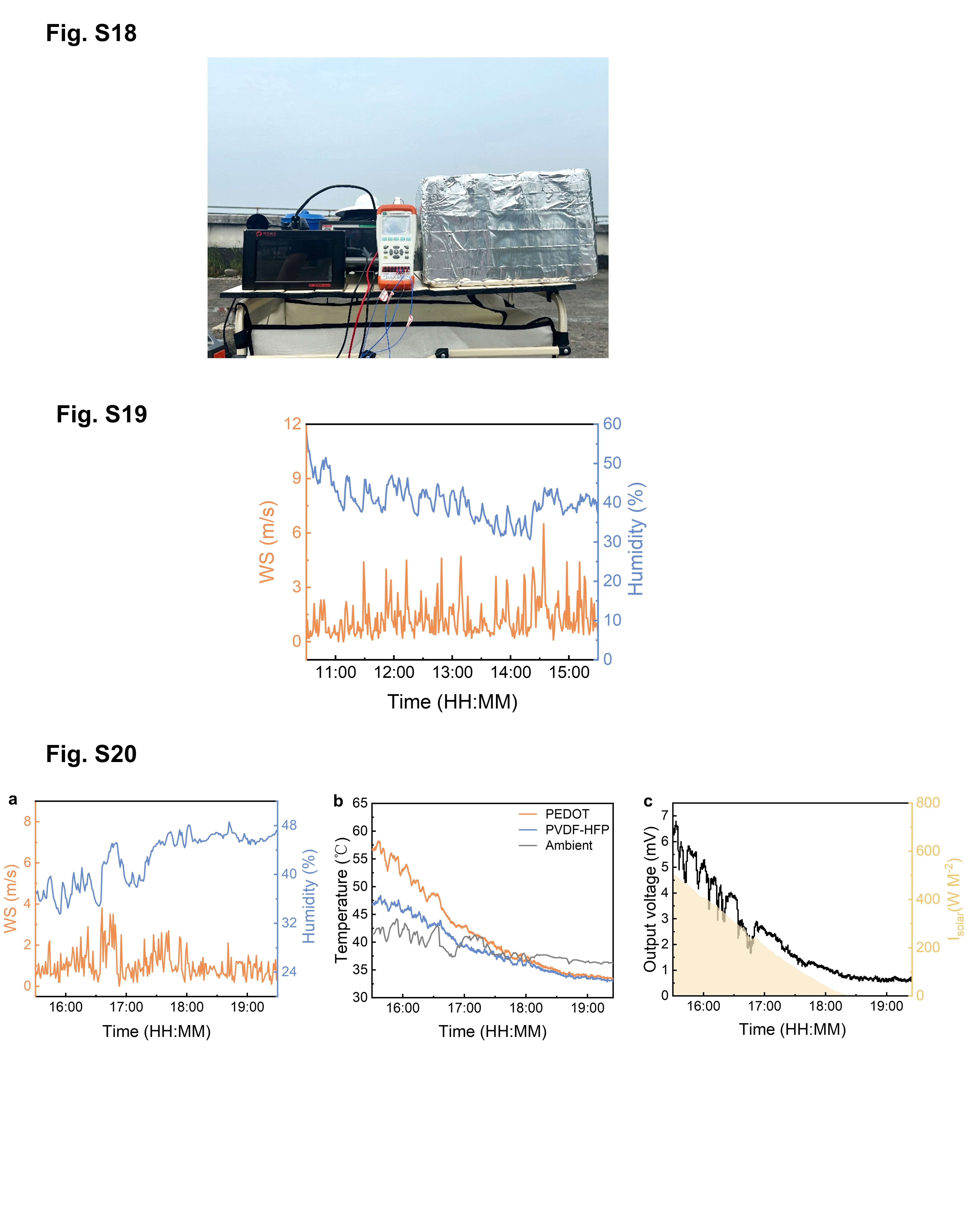


**Fig. S24** Digital photograph of the outdoor experimental setup.


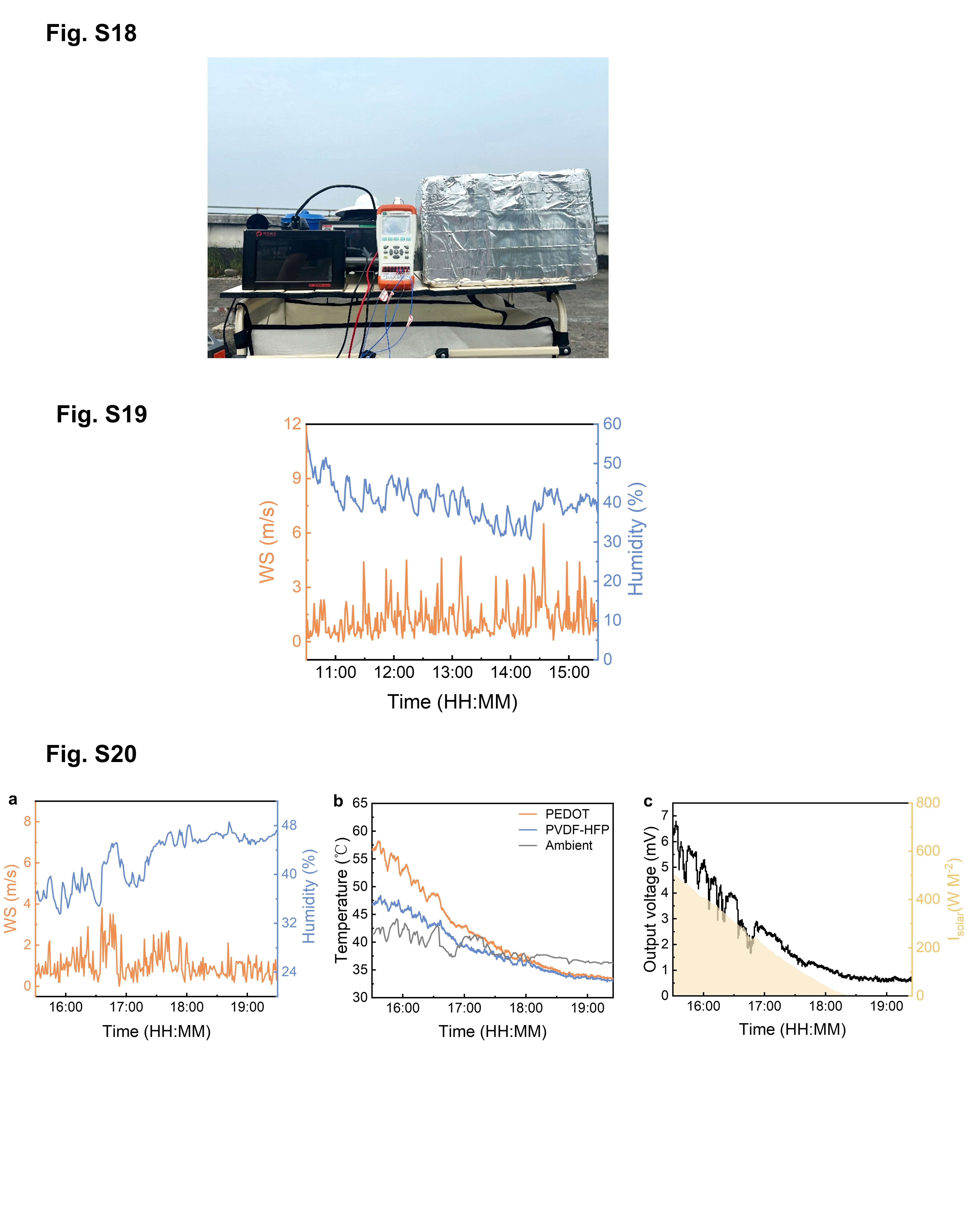


**Fig. S25** Ambient humidity and wind speed during the outdoor test (Suzhou, China, 22 August 2025, 10:30–15:30).


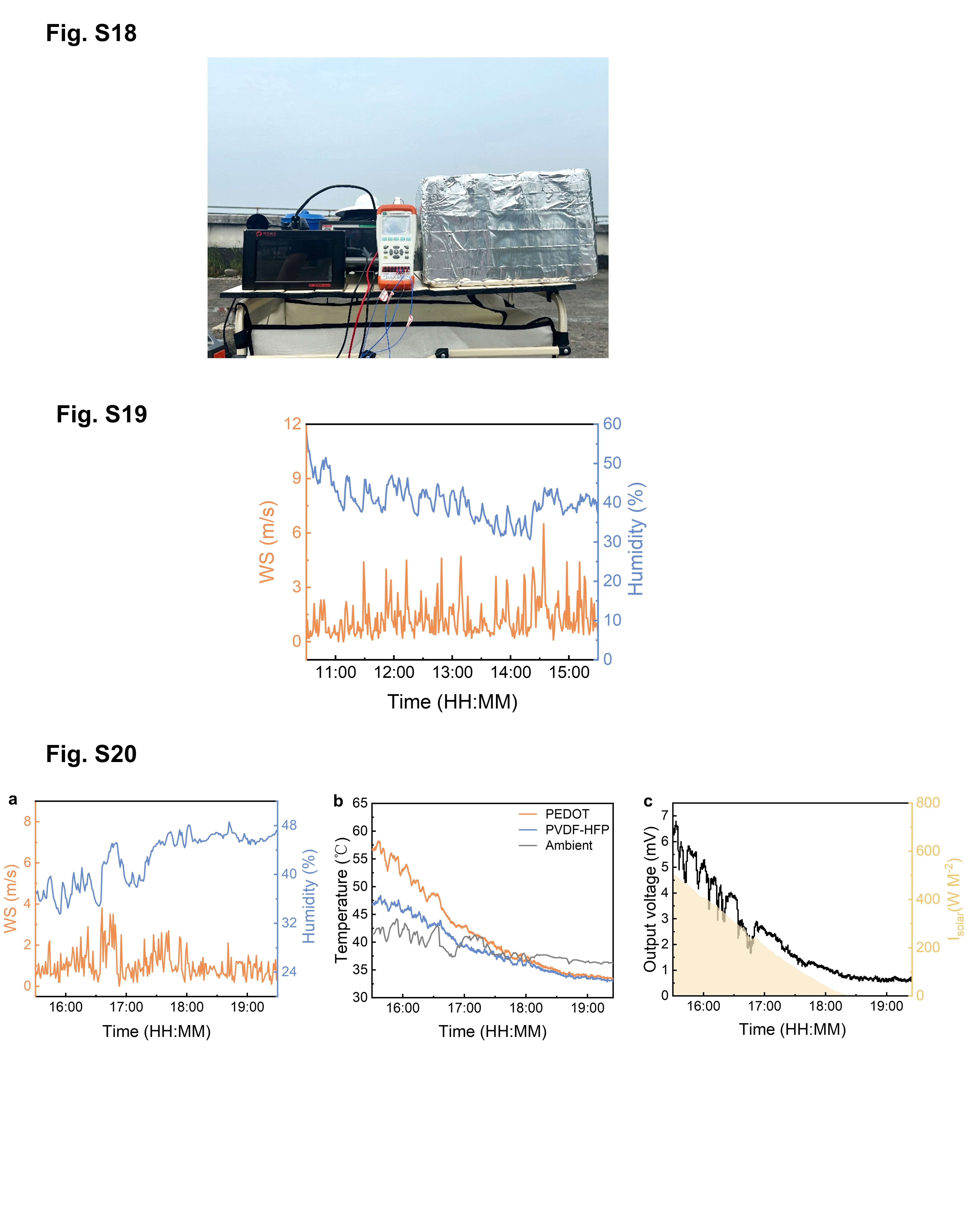


**Fig. S26 a** Ambient relative humidity and wind speed recorded during the test (Suzhou, China, 22 August 2025, 15:30–19:30). **b** Comparative temperatures of the ambient environment, PVDF-HFP nanofiber membrane, and PEDOT:PSS fabric during outdoor testing. **c** Real-time output voltage generated by the radiation-modulated planar fabric.

**
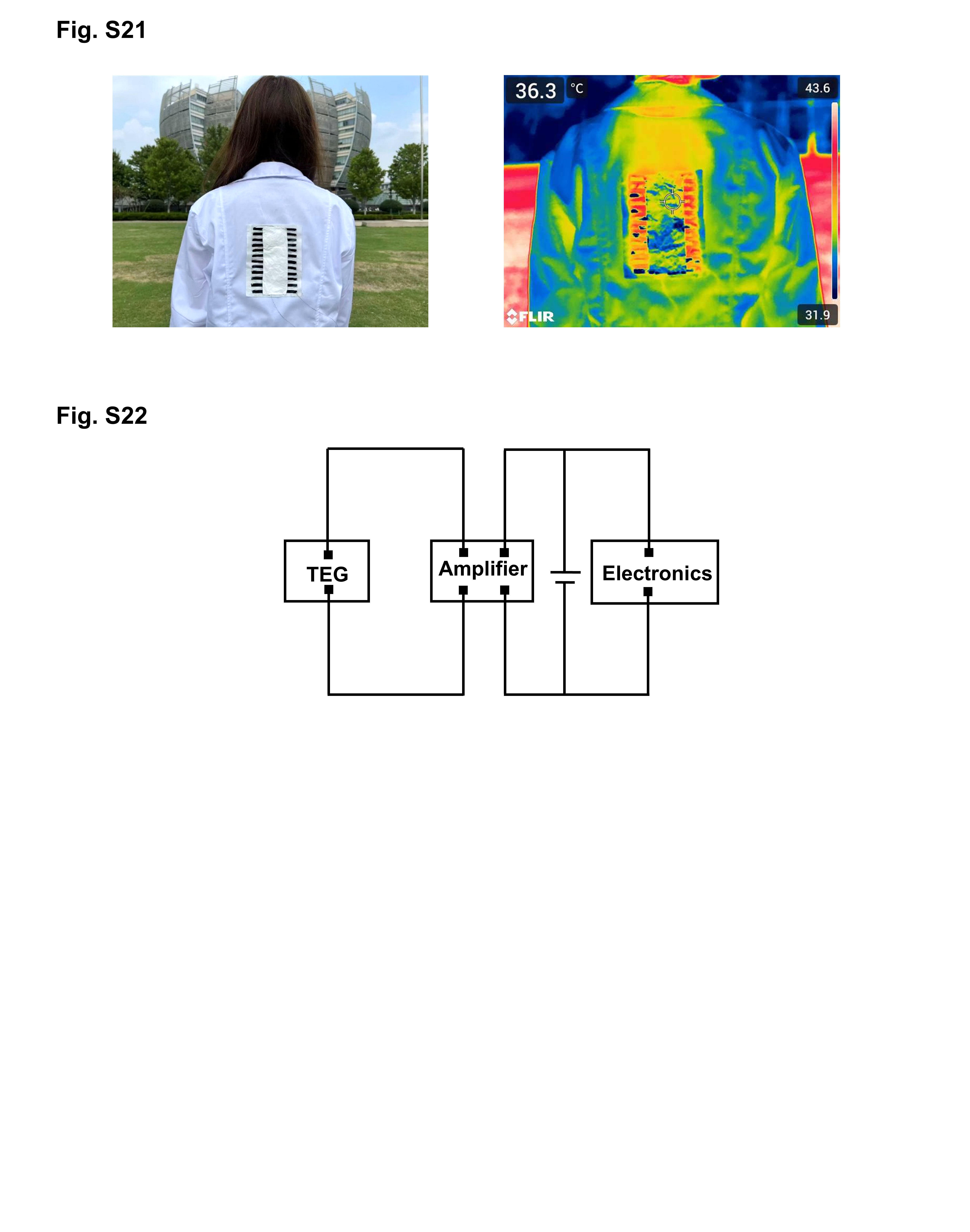
**

**Fig. S27** Photograph of the radiation-modulated planar fabric worn on the body and corresponding infrared image.


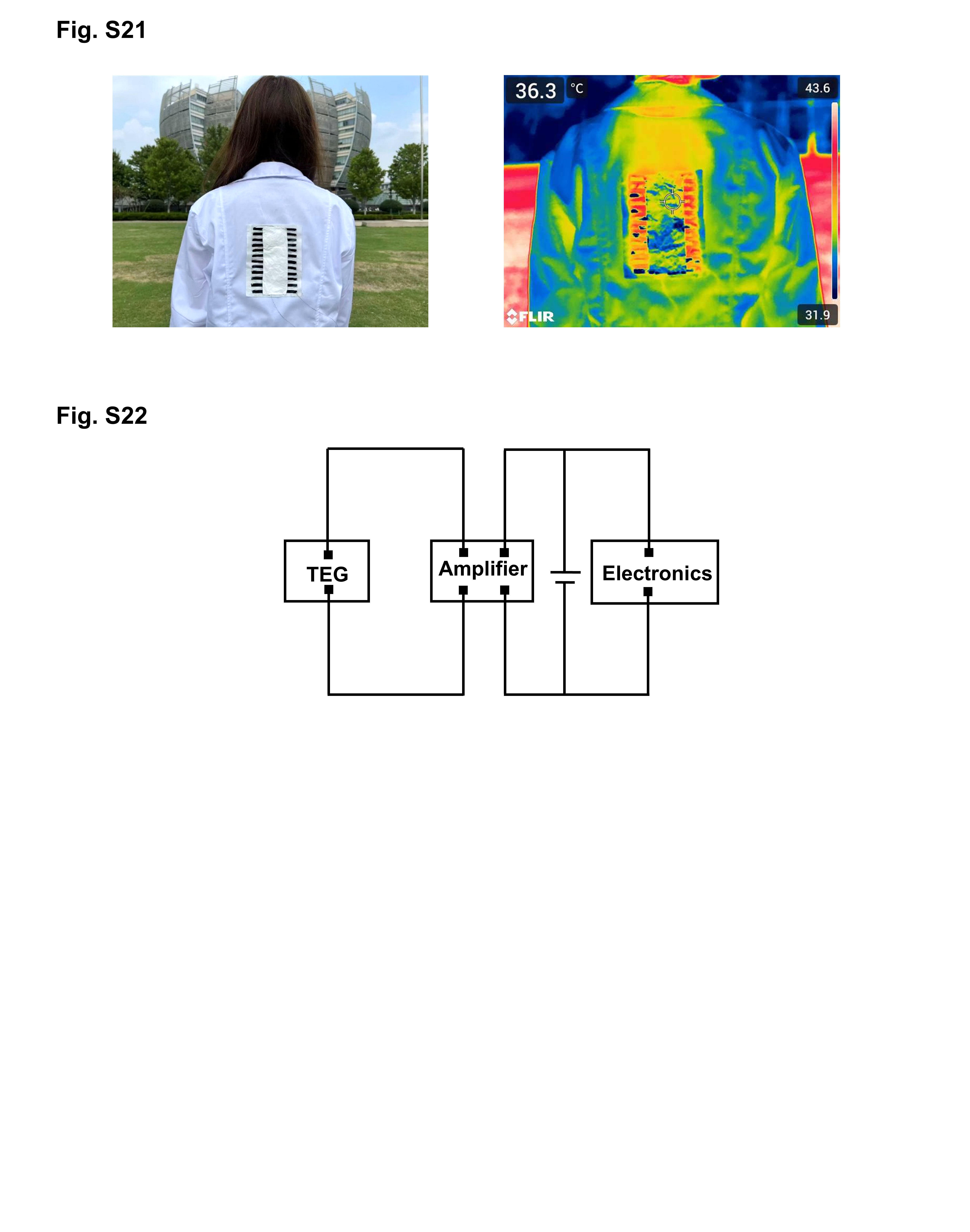


**Fig. S28** Circuit diagram for intermittent LED operation. The TEG fabric is connected to an LTC3108 boost converter, which charges a 0.1 F supercapacitor (2.7 V). When the capacitor voltage reaches 2.2 V (stored energy ~0.24 J), the output is switched to an LED display (28 mW, 10 mA), illuminating it for approximately 0.5 seconds before the voltage drops below the hold threshold. The remaining energy allows additional short pulses. The long charging time (several hours) reflects the low average power of the TEG under outdoor conditions, but the concept of energy storage for intermittent operation is validated.

**

**Fig. S29** Interfacial adhesion strength of the PVDF-HFP/PEDOT:PSS bilayer structure: initial, after 7-day ambient storage, and after 500 bending cycles.

**Table S1** Microfluidic parameters and corresponding shear rates

| **Shear rate (s^-1^)** | **Relative velocity (mm s^-1^)** | **Channel inner diameter (mm)** | **Viscosity (Pa·s)** |
| --- | --- | --- | --- |
| 56 | 33.6 | 0.6 | 0.041 |
| 140 | 86.4 | 0.6 | 0.033 |
| 230 | 138 | 0.6 | 0.029 |

Note: The shear rate was calculated assuming non-Newtonian fluid behavior and no-slip boundary conditions at the channel walls.

**Table S2** Comparison of thermoelectric performance between microfluidic-spun PEDOT:PSS fibers and other reported organic fibers and films

| **Materials** | **Treatment** | ***σ***  **(S cm^−1^)** | | ***S***  **(μV K^−1^)** | | **PF**  **(μW m^−1^ K^−2^)** | | **Measurement conditon** | | **Ref.** | |  |
| --- | --- | --- | --- | --- | --- | --- | --- | --- | --- | --- | --- | --- |
| PEDOT:PSS^a^/DMSO | / | | 1013 | | 19 | | 34 | | Room temperature | | [53] | |
| PEDOT:PSS^a^/DMSO | H_2_SO_4_ | | 4464.6 | | 13.5 | | 80.8 | | Room temperature | | [22] | |
| PEDOT:PSS^a^/DMSO | H_2_SO_4_ | | 4029.5 | | 19.2 | | 147.8 | | Room temperature | | [47] | |
| PEDOT:PSS^a^/DMSO | H_2_SO_4_+NaBH_4_+EMIM:DCA | | 1395.5 | | 19.9 | | 55.4 | | Room temperature | | [44] | |
| PEDOT:PSS^a^/MMIM:DCA | H_2_SO_4_+NaBH_4_ | | 1281.3 | | 26.7 | | 91.7 | | Room temperature | | [54] | |
| PEDOT:PSS^b^/DMSO | DMSO+Na_2_SO_3_ | | 835 | | 35.5 | | 105.2 | | / | | [55] | |
| PEDOT:PSS^b^ | MSA+NaOH+  DMSO | | 1465 | | 42.7 | | 267 | | / | | [36] | |
| PEDOT:PSS^b^ | VC | | 411.2 | | 35.5 | | 51.8 | | Room temperature | | [56] | |
| PEDOT:PSS^b^ | Zn(TFSI)_2_ | | 892 | | 26 | | 60.3 | | Room temperature | | [16] | |
| PEDOT:PSS^b^ | H_2_SO_4_+water+  ethanol solution of TDAE | | 1552 | | 58.2 | | 526 | | / | | [21] | |
| PEDOT:DS^b^ | EMIM:DCA | | 568 | | 38.4 | | 84 | | Room temperature | | [57] | |
| P3HT^b^ | AuCl_3_ | | 207 | | 73.9 | | 110 | | Room temperature | | [58] | |
| PEDOT:PSS^a^/DMSO | H_2_SO_4_+NaOH | | 2037.8 | | 30.4 | | 188.3 | | Room temperature | | **This work** | |

^a^ Fiber; ^b^ Film
